# Impaired β-arrestin recruitment and reduced desensitization by non-catechol agonists of the D1 dopamine receptor

**DOI:** 10.1038/s41467-017-02776-7

**Published:** 2018-02-14

**Authors:** David L. Gray, John A. Allen, Scot Mente, Rebecca E. O’Connor, George J. DeMarco, Ivan Efremov, Patrick Tierney, Dmitri Volfson, Jennifer Davoren, Edward Guilmette, Michelle Salafia, Rouba Kozak, Michael D. Ehlers

**Affiliations:** 1 Medicine Design, Pfizer Worldwide Research & Development, Cambridge, MA 02139 USA; 2 Internal Medicine, Pfizer Worldwide Research & Development, Cambridge, MA 02139 USA; 3 Medicine Design, Pfizer Worldwide Research & Development, Groton, CT 06340 USA; 4 Comparative Medicine, Pfizer Worldwide Research & Development, Cambridge, MA 02139 USA; 5 Present Address: Biogen, Inc., 225 Binney St., Cambridge, 02142 MA USA; 6 Present Address: University of Texas Medical Branch, 301 University Boulevard, Galveston, TX 77555 USA

## Abstract

Selective activation of dopamine D1 receptors (D1Rs) has been pursued for 40 years as a therapeutic strategy for neurologic and psychiatric diseases due to the fundamental role of D1Rs in motor function, reward processing, and cognition. All known D1R-selective agonists are catechols, which are rapidly metabolized and desensitize the D1R after prolonged exposure, reducing agonist response. As such, drug-like selective D1R agonists have remained elusive. Here we report a novel series of selective, potent non-catechol D1R agonists with promising in vivo pharmacokinetic properties. These ligands stimulate adenylyl cyclase signaling and are efficacious in a rodent model of Parkinson's disease after oral administration. They exhibit distinct binding to the D1R orthosteric site and a novel functional profile including minimal receptor desensitization, reduced recruitment of β-arrestin, and sustained in vivo efficacy. These results reveal a novel class of D1 agonists with favorable drug-like properties, and define the molecular basis for catechol-specific recruitment of β-arrestin to D1Rs.

## Abstract (web summary)

Dopamine receptor agonists are used for the treatment of various psychiatric diseases. Here, the authors screen approximately three million compounds and identify a novel class of D1R agonists that do not have a catechol scaffold and possess promising pharmacokinetic properties.

## Introduction

Dopamine is a fundamental catecholamine neurotransmitter, acting via five G protein-coupled receptors (GPCRs) to modulate diverse functions^1^. In the brain, the D1 dopamine receptor (D1R) is highly expressed in the striatum and prefrontal cortex (PFC) where it plays a central role in synaptic plasticity, basal ganglia-mediated motor control^2^, motivation^3^, top-down executive control^4^, learning^5^, and memory^6^. Inadequate dopaminergic neurotransmission in corticostriatal pathways is the demonstrated^7,8^, or hypothesized^9–13^, pathophysiologic underpinning of disabling neurologic and psychiatric illnesses including Parkinson's disease (PD), attention deficit hyperactivity disorder (ADHD), cognitive impairment in schizophrenia, and addiction. As such, selective agonism of D1Rs has long been pursued as a putative therapeutic strategy^14–16^.

Development of clinically effective, selective D1R agonists for central nervous system (CNS) disorders has been limited by the dependence on a characteristic dopamine-like, dihydroxyphenyl catechol structural element^14,17,18^. Mutagenesis studies, as well as recent crystal structures of the related β2-adrenergic receptor (β2AR) bound to catecholamine agonists, have demonstrated tight hydrogen bond associations between the catechol hydroxyl group and serine residues on transmembrane domain 5 (TM5), supporting the notion that the catechol chemical structure makes essential interactions for driving selective D1R agonism^19,20^. However, as a chemical moiety, catechols have poor CNS penetration, negligible oral bioavailability, and are rapidly metabolized^13,21,22^ via oxidative and conjugation processes including methylation, glucuronidation, and sulfation^23^. These undesirable properties have prevented the therapeutic development of selective D1R agonists, despite significant effort for nearly 40 years^13,24^.

**Fig. 1:**
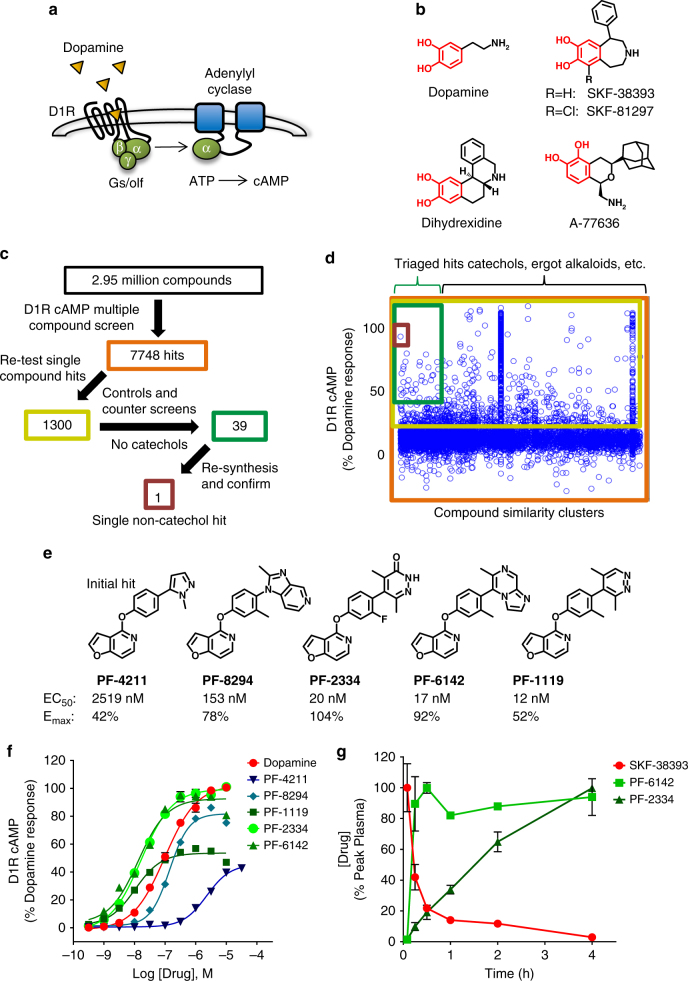
Identification of a novel class of non-catechol D1 dopamine receptor agonists. **a** Schematic of canonical D1 dopamine receptor (D1R) signaling resulting in G_s/olf_ activation and cAMP production. **b** Chemical structures of dopamine and several D1R agonists which contain a catechol moiety (red) limiting drug-like properties. **c**, **d** High-throughput screening (HTS) process flow where 2,953,849 compounds were tested and data representation of D1R agonist activity for all 7748 initial hit compounds (orange box) from the HTS screen grouped by structurally similar ligand clusters, and the triage through 1300 compounds that were re-tested (yellow box), the 39 that passed all filters and counter-screening (green box), which led ultimately to the single validated non-catechol agonist hit, PF-4211 (dark red box). **e** Structure, potency (EC_50_), and intrinsic activity (E_max_) of the non-catechol D1R agonist PF-4211 and several substituted derivatives. **f** Plotted dose-response curves measuring the agonist-induced increases in cAMP levels; *n = *12 per data point. **g** Pharmacokinetic analysis of peak serum drug concentration in rats detected after 5 mg/kg oral administration. Non-catechol agonists had variable kinetics of gut absorption, but displayed a sustained plasma concentration profile compared to the prototypical catechol D1R agonist SKF-38393. SKF-38393 *n = *3; PF-6142, PF-2334 *n = *2. All data indicate means ± s.e.m.

Binding of dopamine to the D1R induces conformational changes which drive guanine nucleotide exchange on heterotrimeric G_s_ or G_olf_ proteins that stimulate adenylyl cyclase and increase intracellular cAMP (Fig. 1a)^1^. Canonical agonist activation of the D1R leads to phosphorylation of receptor intracellular domains by G protein coupled receptor kinases (GRKs) and other kinases^25,26^, which triggers the translocation and coupling of β-arrestins and receptor endocytosis^27^. With prolonged exposure to endogenous or exogenous agonists, β-arrestin association and endocytosis of GPCRs manifests as a desensitization of the pharmacological response in vitro^28^ and tachyphylaxis in vivo^29,30^, phenomena that have further limited advancement of D1R agonists as pharmacotherapeutics^16,31–37^.

Here we report the discovery of a novel series of highly selective, non-catechol D1 receptor agonists with excellent in vivo pharmacokinetic properties. Using in vitro pharmacology and cell-based imaging, we show that these non-catechol ligands display reduced recruitment of β-arrestin and significantly less receptor desensitization compared to catechol agonists, and link these effects result to a novel D1R binding interaction, establishing a putative structural underpinning for agonist functional selectivity at the orthosteric site of a GPCR. An exemplar non-catechol demonstrated continuous sustained behavioral activity in vivo over three days in the rat 6-OHDA unilateral lesion model of Parkinson′s disease, suggesting that compounds from this series may hold therapeutic promise in neurologic and psychiatric diseases involving impaired or reduced dopaminergic signaling.

## Results

### A novel series of non-catechol D1R agonists

To identify D1R agonists which lacked the stereotypical dihydroxyphenyl catechol moiety common among dopamine and all known selective D1R agonists such as A-77636, dihydrexidine, SKF-81297, and SKF-38393 (Fig. 1b), we conducted a high-throughput screen. Compounds curated for drug-like properties^38^ were screened for their ability to increase intracellular cAMP in a stable HEK293 cell line expressing high levels of D1R (0.593 pmol/mg protein). Assay conditions were optimized specifically to detect low potency and low intrinsic activity hits such that known partial agonists like SKF-38393 (Fig. 1b) appeared as full agonists. Of 2,953,849 compounds screened, 1300 produced a percent elevation of cAMP greater than three standard deviations above the mean and were confirmed when re-tested as single compounds at 20 μM (Fig. 1c). Conducting the cAMP assay in a non-D1R-expressing parental HEK cell line revealed that many of the hits were acting independent of the D1R. Subsequent compound triage was conducted to remove artifactual and undesirable compounds including those that exhibited more than 20-fold preference for D2R or contained specific structural elements (ergot alkaloids, catechols, phenols, dopamine derivatives). This iterative filtering left 39 potential “hits” (Fig. 1c), which were individually re-synthesized and thoroughly investigated. A majority of hits were eliminated because they either showed no dose response, had activity traced to screening sample impurities with known pharmacology (e.g., dopamine), or elevated cAMP via non-D1R mechanisms (e.g., phosphodiesterase (PDE) inhibition). Ultimately, only one compound, termed PF-4211, remained from the screening process as a true non-catechol D1R-preferring agonist (Fig. 1c, d).

PF-4211 exhibited modest potency (EC_50_ = 2519 nM) and partial agonism (*E*_max_ = 42%) at the D1R. The expected basic amine functionality was lacking from PF-4211, which features a linear triaryl structure that is notably longer and more rod-like than other known D1R agonists (Fig. 1b, e). Beginning with this lone low potency hit, we modified each of the three main structural sections in an iterative process of design, chemical synthesis, and screening of numerous compounds to establish relationships between structure and D1 activity. Replacement of the N-methyl pyrazole terminus with specific heterocyclic moieties such as imidazopyridine, pyrazine, pyrazinone, and imidazopyrazine improved CNS drug-like properties^39,40^ while also increasing D1R activity (Fig. 1e). Substitutions adjacent to the junctional bond between the central phenyl and terminal rings increased potency, and suggested that increased steric demand about the aryl–aryl bond enforces conformational constraints favorable for D1R affinity. The intrinsic activity of analogs varied from partial to full D1R agonism when switching among several heterocycles (Fig. 1e, f). After significant optimization, several compounds displayed D1R potency in the low-nM range (Fig. 1e, f), similar to that of the most potent catechol D1R agonists (Fig. 1b, Supplementary Table 1). These compounds were highly selective for the D1R-like class (D1R and D5R) of dopamine receptors with minimal binding to D2, D3, and D4 dopamine receptors; they were also selective against a broad CNS selectivity panel of 30 GPCR′s, enzymes, and ion channels, including no detectable interaction with or agonism of the 5HT2B receptor (Supplementary Tables 1 and 2). No selective agonist ligands were identified which significantly discriminated between the highly homologous D1 and D5 receptor subtypes^41^, and thus for simplicity, we refer to all of these compounds as D1R ligands (Supplementary Table 1). Pharmacokinetic studies in rats showed that the prototypical D1R agonist SKF-38393 was characterized by low oral bioavailability and rapid clearance after oral administration as expected for the catechol class^23^. In contrast, non-catechol agonists PF-6142 and PF-2334 displayed more sustained plasma concentration profiles with varying rates of gut absorption (Fig. 1g and Supplementary Table 3). Thus, deep mining of a large chemical file together with an iterative medicinal chemistry and screening effort led to a structurally novel class of potent, selective, orally bioavailable, and metabolically stable non-catechol D1R agonists.

### Non-catechol agonists do not desensitize D1Rs

**Fig. 2:**
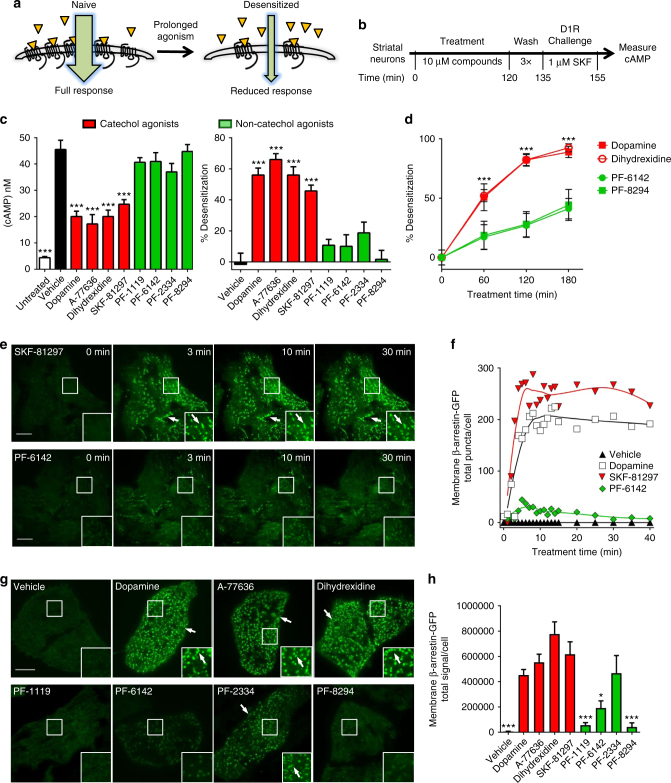
Non-catechol D1R agonists fail to induce desensitization via impaired β-arrestin recruitment. **a** Schematic of agonist-stimulated desensitization of GPCRs. Prolonged activation reduces subsequent agonist-induced signaling. **b** Experimental design to test for agonist-stimulated D1R desensitization. **c** Agonist-induced (1 μM SKF-81297) cAMP elevation and percent desensitization of D1 receptor signaling in striatal neurons treated for 120 min with vehicle or 10 μM of the indicated agonists. One-way ANOVA revealed a significant effect of treatment on cAMP levels, *F*(9, 108) = 32.1, *P* < 0.001; post-hoc Dunnett’s comparisons indicated only catechol agonists had significantly different level of cAMP versus vehicle (***P < 0.001). Percent desensitization was significantly affected by treatment (one-way ANOVA *F*(8,99) = 24.2, *P* < 0.0001); Dunnett’s comparisons indicated significantly higher percent desensitization in all catechol groups versus vehicle (***P < 0.001), while non-catechol groups were not significantly different from vehicle. All data represent means ± s.e.m., *n* = 3 per group. **d** Percent D1 receptor desensitization after various times of agonist exposure. Two-way ANOVA revealed significant trends in time (linear *F*(1,59) = 44.2, *P* < 0.0001 and quadratic *F*(1,59) = 8.5, *P* < 0.005) and significant interaction effect (*F*(3,59) = 5.1, *P* < 0.003); pairwise Tukey post-hoc tests at time points 60,120, and 180 min indicated that catechol and non-catechol groups were significantly different (***P < 0.001, *n = *4 per group; batches are independent from panel **c**). **e** Live cell total internal reflection fluorescence microscopy (TIRFM) imaging of β-arrestin-GFP recruitment to the plasma membrane of U2OS cells expressing human D1R. Images show β-arrestin-GFP at the plasma membrane of cells before and after exposure to the indicated agonists (1 μM). **f** Quantification of membrane β-arrestin-GFP puncta in the cells shown in **e**. **g** TIRFM images of β-arrestin-GFP at the plasma membrane in U2OS cells after 10 min of treatment with indicated agonists (1 μM). Scale bars, 10 μm. **h** Quantification of β-arrestin-GFP membrane recruitment via total signal per cell from ≥60 cells per group obtained across three independent experiments. One-way ANOVA revealed that treatment had a significant effect (*F*(9,33) = 130.9, *P* < 0.0001; Dunnett’s comparisons with dopamine group indicated significantly lower total signal in vehicle, PF-1119, PF-8294 (***P < 0.001) and PF-6142 (*P < 0.05), but not in PF-2334, and not for all catechol agonists groups. All data represent means ± s.e.m., *n* ≥ 3

Receptor desensitization in response to prolonged agonist exposure is a hallmark feature of many GPCRs^28,42^ including D1R′s, and can limit therapeutic potential (Fig. 2a). To examine whether structurally novel non-catechols produce D1R desensitization similar to canonical catechol agonists^25,26,43,44^ in a native system, we pre-exposed rat striatal neurons to control conditions or test compounds for 2 h, washed, and then measured cAMP levels in response to a D1R agonist challenge (1 μM SKF-81297, 20 min) (Fig. 2b). Striatal neurons exhibit low basal cAMP that was significantly increased by SKF-81297 challenge (vehicle in Fig. 2c, left panel). Pre-treatment with catecholamine agonists across several structural subtypes (dopamine, A-77636, dihydrexidine, SKF-38393) blunted the cAMP increase induced by subsequent D1R agonist challenge by 40–60%, consistent with previous findings and indicating pharmacological desensitization (Fig. 2c, d). In contrast, pretreatment with non-catechol agonists (PF-1119, PF-6142, PF-2334, PF-8294) did not diminish the subsequent elevation in cAMP by D1R agonist challenge (Fig. 2c, d).

To test whether the lack of desensitization triggered by non-catechol D1R agonists is due to a failure to recruit β-arrestin^45^, we performed live cell imaging experiments. The human D1R was expressed in U2OS cells stably expressing β-arrestin2-GFP and the appearance of arrestin puncta at the plasma membrane was visualized using total internal reflection fluorescence microscopy (TIRFM). In response to the catechol agonist SKF-81297 (1 μM) or to dopamine (1 μM), membrane associated β-arrestin puncta appeared rapidly at presumptive clathrin-coated pits (Fig. 2e, f; Supplementary Fig. 1a, Supplementary Movies 1–3). However, the potent non-catechol agonist PF-6142 induced significantly less recruitment of β-arrestin (Fig. 2e, f; Supplementary Movie 4). Rapid and robust β-arrestin recruitment was observed with every catechol compound tested, while none of the novel non-catechol D1R agonists elicited such significant β-arrestin relocation to plasma membrane puncta (Fig. 2g, h). Taken together, these results indicate that potent D1R agonists lacking the dihydroxyphenyl catechol moiety stimulate G protein-mediated cAMP signaling, but fail to fully trigger β-arrestin recruitment and associated desensitization, suggesting a differential mode of functional agonism and binding.

### A unique binding mode for non-catechols at the D1R

**Fig. 3:**
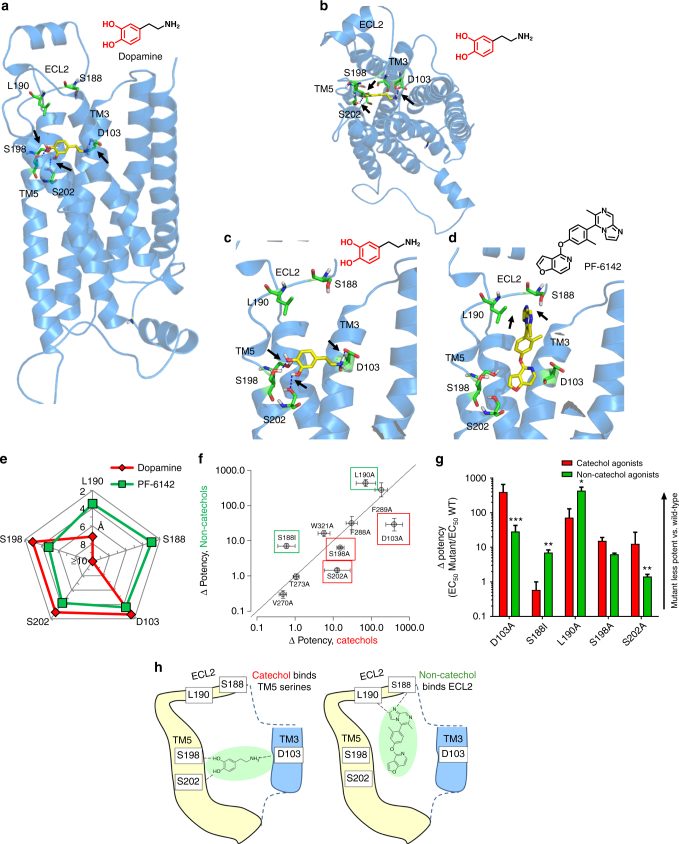
Non-catechol D1R agonists adopt a unique binding mode contacting ECL2. **a**–**e** Structural homology ligand docking models of the human D1R were simulated using the agonist bound crystal structure of the β2 adrenergic receptor. **a**–**c** Docking models of dopamine bound to the D1R in the orthosteric binding site show catechol hydroxyls making hydrogen bond polar interactions with serines 198 and 202 (S198, S202) on transmembrane helix 5 (TM5) and the amine group forming a salt bridge ionic interaction with glutamate 103 (D103) on helix 3 (TM3). **d** Non-catechol agonist PF-6142 modeled in the orthosteric site and extended D1R binding pocket and making notable contacts with residues in extracellular loop 2 (ECL2) including serine 188 (S188) and leucine 190 (L190). **e** Molecular docking computations showing the nearest atomic distance (in Å) between dopamine or PF-6142 and key amino acid residues in the D1R orthosteric and extended sites as a spider plot. PF-6142 displayed close atomic juxtaposition to S188 and L190 within ECL2 and more distant positioning relative to residues in TM5. **f** Correlation plot of catechol and non-catechol agonist potency shifts in functional assay (Δpotency = D1 cAMP EC_50mut_/D1 cAMP EC_50wt_) caused by mutation of individual D1R residues. Mutation of ECL2 residues S188A and L190A (green boxes) resulted in larger Δpotency for non-catechol agonists while mutation of TM5 S198A or S202A and TM3 D103A (red boxes) selectively reduced catechol agonist potency. **g** Change in potency of catechol or non-catechol agonist-induced cAMP signaling following mutation of critical residues identified by docking models. Data are presented as Δpotency on a log scale defined as the potency means ± s.e.m. of mutant/wild type receptor. Two-way ANOVA revealed significant effects of mutant (*F*(4,34) = 29.7, *P* < 0001 and interaction between mutant and compound type (*F*(4,34) = 14.7, *P* < 0001, non-significant effect of compound type (*F*(1,34) = 2.9, *P* < 0.1, post-hoc tests indicated significantly higher Δpotency in catechol group for D103A, S202A (***P < 0.001) and lower Δpotency in catechol group for S188I (***P < 0.001) and L190A (*P < 0.05). **h** Schematic model of dopamine or PF-6142 differentially binding to TM5, TM3, and ECL2 residues surrounding the D1R orthosteric ligand binding site

Intrigued by their novel functional profile and lack of basic amine and polar hydroxyl catechol functionalities (Fig. 1), we sought to gain insight into the binding mode of non-catechol agonists. The crystal structure of the D1R has not been solved; however, the β2 adrenergic receptor (β2AR) has a similar binding site to D1R^46^ and recent studies have described high resolution structures of the agonist-bound β2AR. We previously computed a structural homology model of the human D1R^20^ based on atomic coordinates of the agonist-bound β2AR (PDB accession code: 3P0G) using binding and functional data from available catechol ligands^47^. Consistent with crystal structure and site-directed mutagenesis data, computational docking and energy minimization of dopamine into the orthosteric ligand binding site of this model projects a key ionic salt bridge interaction between the primary amine and aspartate 103 (D103) on transmembrane 3 (TM3), and close hydrogen bond contacts with serines 198 and 202 (S198, S202) on transmembrane 5 (TM5) (Fig. 3a–c; Supplementary Movie 5). The docking of other catecholamine D1R ligands identified similar preferred contacts at the D1R orthosteric site^20^. Building on these results, computational docking of non-catechol agonists such as PF-6142 into the same D1R model indicated energetically favorable binding poses within the orthosteric ligand binding site that lacked direct interaction with D103 in TM3 and showed diminished contacts with S198 and S202 in TM5 (Fig. 3d). These docking orientations predict that the loss of energetically favorable interactions with TM3 and TM5 is offset by favorable receptor contacts that extend toward the extracellular face including previously undescribed interactions with S188 and L190 on ECL2 (Fig. 3d; Supplementary Movie 6). With slight variation according to the heterocycle, this extended binding configuration and interaction with ECL2 was common to modeled poses for all non-catechol agonists examined (Supplementary Movies 6–8).

To estimate the significance of the interaction between different ligands and specific D1R residues of interest, we computed the nearest atomic distance between the agonist and select amino acid residues after docking and energy minimization within the orthosteric site. This analysis predicted close atomic proximity (<3 Å) of dopamine to S198, S202, and D103 and no proximity to S188 and L190 (Fig. 3e). In contrast, non-catechol PF-6142 was projected to bind with close atomic juxtaposition to S188 and L190 within ECL2 but increased distance to residues in TM5 (S198, S202) and TM3 (D103) (Fig. 3e, Supplementary Table 4). Thus, as a class, bound catechol D1R agonists are predicted to associate closely with serine residues in TM5 and form a close ionic contact with TM3, while non-catechol agonists interact strongly with ECL2, providing a potential biophysical basis for differential desensitization and β-arrestin recruitment.

To provide experimental support for the modeling results and distinct binding poses computed for catechol and non-catechol agonists, we first looked to confirm the general orthosteric positioning of the binding site for non-catechol agonists. In competition binding experiments conducted in the presence of increasing concentrations of established orthosteric D1 antagonist SCH-23390, PF-6142 showed fully competitive kinetics at low concentrations, while data from two other ligands in the series were consistent with a mixed profile of both competitive and non-competitive kinetics (Supplementary Table 5, Supplementary Figures 2 and 3). We then measured the effect of targeted single residue D1R mutations on cAMP signaling in response to stimulation by catechol or non-catechol agonists. We reasoned that mutation of residues selectively involved in catechol or non-catechol binding would preferentially attenuate the functional potency across compounds of the corresponding agonist class. The impact of each mutation for each compound was expressed as a change in cAMP EC_50_ relative to the cAMP EC_50_ at the wild type D1R (Δpotency = EC_50mut_/EC_50wt_) such that values >1 indicate diminished cAMP response at the mutant receptor relative to wild type. For overall analysis, we pooled data testing the effect of D1R mutations on the potency of ten catechol agonists and 18 non-catechol agonists. Plotting mean Δpotency for the two ligand classes across ten selected mutants revealed those that preferentially impact the potency of one agonist class (Fig. 3f). Additional mutations were tested and showed no preferential effect on potency or efficacy between the two compound classes (Supplementary Table 6). Mutation of D103 within TM3 to alanine consistently resulted in very large loss of activity (Δpotency > 100) for all catechols, but had only modest impact on agonist potency across the series of non-catechols (Fig. 3g), consistent with the formation of a salt bridge contact between the amino group only present in the catechols and the carboxyl moiety of D103 (Fig. 3a–c, e, h). In addition, mutation of S198 or S202 within TM5 to alanine resulted in a larger Δpotency in response to catechol agonists relative to non-catechols (Fig. 3g), as predicted by our computational model (Fig. 3a–d). On the other hand, mutation of S188 to isoleucine and L190 to alanine within ECL2 conferred a much larger Δpotency for non-catechol agonists relative to catechols (Fig. 3f, g). This series-level difference was highly significant with D103A, S198A, S202A mutations having more impact on catechol agonism, and S188I mutations showing more impact on non-catechol agonism when comparing Δpotency for all compounds in a class. Together, these findings coalesce to support a model for distinct modes of catechol and non-catechol binding within the broadly defined D1R orthosteric site (Fig. 3h).

### Hybrid with catechol reintroduced desensitizes D1Rs

**Fig. 4:**
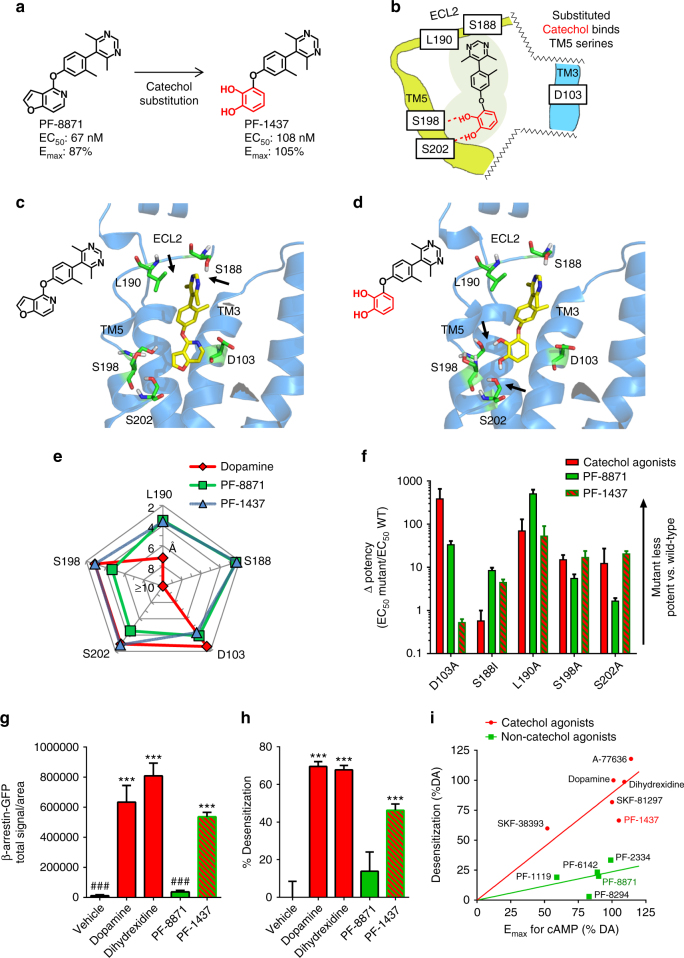
Re-introducing catechol restores serine interactions, and β-arrestin recruitment. **a** Chemical structures, potency, and efficacy of the non-catechol agonist PF-8871 and its corresponding substituted catechol isostere PF-1437. **b** Schematic model of the hybrid catechol PF-1437 binding to TM5 serine residues and remaining in contact with extracellular loop 2 (ECL2). **c** Homology docking model of the non-catechol agonist PF-8871 bound in the orthosteric and extended D1R binding pocket making contacts with ECL2 residues S188 and L190. **d** Homology docking model of the catechol converted analogue PF-1437 bound to the D1R showing predicted catechol hydroxyl hydrogen bond polar interactions with S198 and S202 on transmembrane helix 5 (TM5). **e** Spider plot of calculated atomic proximity (Å) of ligands relative to important residues in TM5, TM3, and ECL2 from molecular docking simulations. **f** Change in cAMP signaling potency of dopamine, PF-8871, and the hybrid catechol PF-1437 upon mutation of select D1R residues. Δpotency (mutant/wildtype); for dopamine, PF-8871, and PF-1437 (*n* ≥ 3). **g** Quantification of agonist-stimulated β-arrestin-GFP recruitment to the plasma membrane. Data represent total signal per cell measured by TIRFM from ≥60 cells per group, (*n* ≥ 3). Square root transformation was used to stabilize variance (Bartlett’s test, *P* > 0.2; one-way ANOVA revealed effect of treatment *F*(5,15) = 210.7, *P* < 0.001; post-hoc comparisons, corrected for multiple testing via FDR, indicated significantly higher signal in dopamine, dihydrexidine, and PF-1437 groups (***P < 0.001) and, in vehicle and PF-8871, significantly lower signal than in dopamine groups (^###^*P* < 0.001). **h** Percent desensitization of D1R signaling in striatal neurons treated for 120 min with vehicle or 10 μM agonists. *n = *3 per group. Percent desensitization was significantly affected by treatment (one-way ANOVA *F*(4,54) = 56.5, *P* < 0.001); Dunnett’s comparisons with vehicle group indicated significantly higher percent in dopamine, dihydrexidine, and PF-1437 groups (***P < 0.001), but not in PF-8871 group. **i** Correlation analysis of percent receptor desensitization (10 μM, 120 min pretreatment) versus maximal efficacy to increase cAMP (E_max_). All agonist responses were normalized to percent dopamine (DA). Pearsons correlation analysis; catechol agonists *r* = 0.890, *R*^2^ = 0.793, *P* = 0.043; non-catechol agonists *r* = 0.293, *R*^2^ = 0.086, *P = *0.663. All data are presented as mean ± s.e.m.

To test whether hydrogen bonding between catechol hydroxyls and TM5 serine residues is involved in β-arrestin recruitment and D1R desensitization, we engineered a hybrid chemical probe where catechol functionality was strategically re-introduced into the novel chemical scaffold (Fig. 4a, b). PF-8871 is a potent and selective non-catechol full D1R agonist featuring a C2-symmetric dimethylpyrimidine that reduces rotational conformations and simplifies computational docking (Fig. 4a). From a comparison of its predicted positioning within the homology model (Fig. 4c, Supplementary Movie 8) relative to docked catechols (Fig. 3a–c), we designed and synthesized an isosteric substitution for the azabenzofuran heterocycle of PF-8871 to produce the hybrid compound PF-1437 (Fig. 4a) that contains a dihydroxyphenyl predicted to engage S198 and S202 (Fig. 4d). After energy minimization, the hybrid compound PF-1437 docked with characteristics of both catechol and non-catechol D1R agonists, making close contacts to ECL2 residues S188 and L190 as well as predicted hydrogen bonding between the catechol and TM5 serines S198 and S202 (Fig. 4b–e; Supplementary Movie 9). In addition, the stability of both PF-1437 and PF-8871 within in the orthosteric binding site during 50 ns molecular dynamic simulations of the compound-protein complex in a lipid bilayer further supported the proposed binding mode (Supplementary Movies 7, 10, and 11). PF-1437 maintained high potency (EC_50_ = 108 nM), selectivity, and full agonism (E_max_ = 105%) at the D1R, indicating that this catechol substitution onto the novel scaffold did not impair agonist-induced coupling to G_s_ and adenylyl cyclase.

To gain experimental confirmation of the predicted hybrid binding profile of catechol agonist probe PF-1437 at the D1R orthosteric ligand binding site, we compared the impact of single residue D1R mutations on the Δpotency of dopamine, the non-catechol agonist PF-8871, and its hybrid isostere PF-1437. Consistent with the series-level structure-function analysis (Fig. 3), mutation of catechol-contacting TM5 serines 198 or 202 to alanine yielded a large Δpotency for dopamine and for PF-1437, but had minimal effect on cAMP production triggered by the non-catechol PF-8871 (Fig. 4f). As predicted, the sizable Δpotency seen with dopamine for the D103A mutant was not seen with PF-8871 or PF-1437 (Fig. 4f, Supplementary Table 6), consistent with reduced molecular contact of these compounds with D103 (Fig. 4e). The S188I and L190A mutations on ECL2 had a somewhat greater impact on Δpotency for PF-8871 and PF-1437 compared to dopamine (Fig. 4f). Thus, introduction of a catechol moiety into the novel scaffold introduces functionally important agonist contact with serine residues on TM5 without introducing sensitivity to the aspartate on TM5, supporting the proposed binding model of catechol-D1R ligands and non-catechol D1R ligands.

To examine the causal relationship between catechol hydroxyl contact with TM5 serines, β-arrestin recruitment, and receptor desensitization, we compared β-arrestin recruitment and c-AMP desensitization between the non-catechol agonist PF-8871 and its catechol isostere PF-1437 (Fig. 4a). Importantly, both compounds are full D1R agonists with closely matched potency (EC_50_) and intrinsic activity (E_max_) for cAMP production (Fig. 4a), indicating that any difference in desensitization or β-arrestin recruitment cannot be attributed to differential partial agonism or potency. Consistent with our results examining other non-catechols (Fig. 2), PF-8871 (1 μM) induced minimal recruitment of β-arrestin to plasma membrane puncta (Fig. 4g). In sharp contrast, the same concentration of the analog hybrid agonist PF-1437 carrying catechol substitution elicited β-arrestin recruitment profile similar to that of dopamine and other canonical D1R agonist catechols (Fig. 4g). Moreover, PF-8871 (10 μM, 2 h) produced very little desensitization of the cAMP response to a subsequent D1R agonist challenge (Fig. 4g), whereas PF-1437 (10 μM, 2 h) robustly reinstated desensitization of the agonist-triggered cAMP response (Fig. 4h). Examination of a series of D1R agonists revealed a steep, linear relationship between intrinsic agonist activity (*E*_max_ for cAMP) and the degree of desensitization for catechol agonists (Fig. 4i), consistent with previous findings^48^. Non-catechol agonists exhibited markedly less desensitization overall regardless of intrinsic agonist activity (Fig. 4i), indicating that the lack of desensitization is a feature of the series as a whole, and not attributable simply to difference in partial agonism^49^. Notably, conversion of PF-8871 to its cognate catechol hybrid PF-1437 shifted its desensitization-*E*_max_ relationship such that it falls in line with other catechols (Fig. 4i). With the complexity and dynamism of GPCR agonism, there may be additional subtle structural contributions to the observed signaling differences between catechol and non-catechols; however, these results indicate that catechol hydroxyl group contacts with TM5 serines are essential for D1R desensitization, and define a molecular basis for catechol-specific recruitment of β-arrestin.

### Non-catechol D1R agonist has sustained in vivo response

**Fig. 5:**
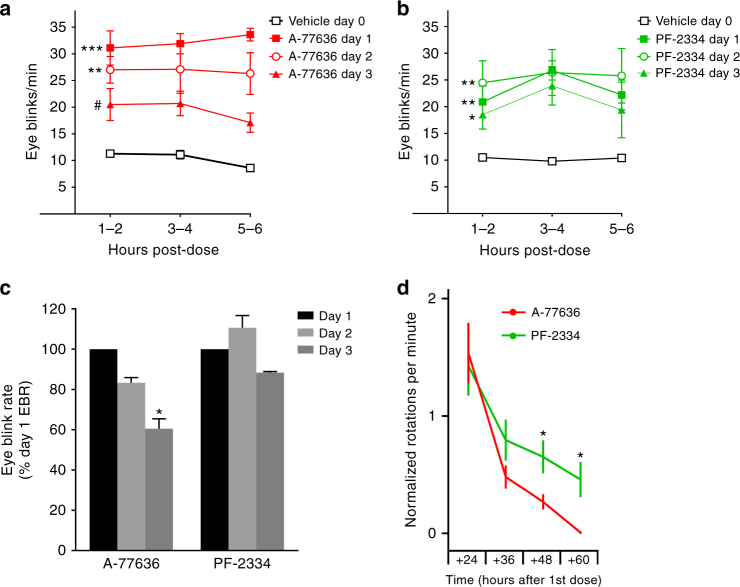
Non-catechol agonists elicit sustained responses after repeated dosing in vivo. Eye blink rates (EBR) in cynomolgus monkeys (*n* = 3) were assessed as a measure of central D1R activation over 6 h after administration of vehicle and repeated administration of agonists daily for 3 days. Dosing of agonists was chosen to give matched D1R occupancy. **a** Monkey eye blink rates over a 6 h period after vehicle or subcutaneous dosing of A-77636 (1 mg/kg). EBR was significantly greater than vehicle group at days 1 (***P = 0.0003) and 2 (*P = 0.0024), but not different for day 3 (*P = *0.065); EBR on day 3 was significantly lower than on day 1 (^#^*P* = 0.011). **b** EBR over 6 h after vehicle or oral administration of PF-2334 (0.6 mg/kg + 0.3 mg/kg 8 h later). EBR was significantly greater than vehicle values for day 1 (**P = 0.010), day 2 (**P = 0.0038), and day 3 (*P = 0.035). EBR on days 1,2,3 showed no pairwise difference. **c** Eye blink rates over the 6 h period after drug administration were combined and are expressed as percent of day one response, *n = *3 per group. **d** Contralateral rotations in rats with confirmed unilateral 6-OHDA lesions after administration of D1R agonists every 12 h. Rotations per minute were measured for the 6 h immediately following subcutaneous doses of A-77636 (0.32 mg/kg, red bars) or oral doses of PF-2334 (10.78 mg/kg, green bars). Two-way ANOVA revealed significant effect of treatment (*P* = 0.022) and time (*P* < 0.0001) but not interaction (*P* = 0.068). After 36 h, PF-2334 treated animals had significantly greater rotational behavior than A-77636 treated animals (*P < 0.05). All data are presented as means ± s.e.m. For full time-course of rotational behavior see Supplementary Fig. 5

Sustained pharmacological activation of D1Rs attenuates subsequent responses in diverse behavioral and physiological paradigms^32,36^. This attenuation, or tachyphylaxis, represents a functional in vivo correlate of receptor desensitization^34^. Although the neuronal circuitry is not well understood, spontaneous eye blink rate (EBR) is considered a functional marker of central dopaminergic activity mediated through D1Rs in both humans and non-human primates^50–52^. We tested whether differences observed in in vitro D1R desensitization (Figs. 2 and 4) would translate to divergent effects of repeated administration between catechol and non-catechol D1R agonists in a cynomolgus monkey model of D1R induced spontaneous EBR (Fig. 5). A-77636 was selected as a well-studied catechol agonist and PF-2334 as a suitably matched non-catechol full agonist based on their comparable D1R potencies, high selectivity versus D2Rs, and pharmacokinetic profiles in non-human primates (Supplementary Tables 7 and 8, Supplementary Fig. 4). Upon subcutaneous administration of a single dose of A-77636 (0.1 mg/kg), spontaneous EBR increased 2–3 fold over baseline and this increase was sustained over several hours (Fig. 5a). With subsequent administrations over several days, the A-77636-induced EBR increase was progressively and significantly attenuated with each dosing day (Fig. 5a, c), indicating tachyphylaxis of an in vivo measure of central dopaminergic activation. In contrast, oral administration of the non-catechol agonist PF-2334 (0.6 mg/kg followed by a 0.3 mg/kg booster dose 8 h later) elicited a similar increase in EBR that was sustained after multiple days of dosing (Fig. 5b, c), with no statistical difference between day 1 and 3.

To explore the therapeutic potential of these novel compounds and further investigate the observed lack of toleration, PF-2334 was tested in the well-established and translationally relevant unilateral 6-hydroxydopamine (6-OHDA) lesion model of Parkinson′s disease^53^. Agents that increase dopamine receptor activation, including effective Parkinson′s therapies, elicit characteristic rotational behavior that is contralateral to the hemisphere that received the lesion and this rotational behavior is reported to desensitize with sustained or repeated drug exposure^34^. Six subcutaneous doses of A-77636, (0.32 mg/kg S.C.) were administered to lesioned rats every 12 h, producing the expected tachyphylaxsis (Fig. 5d, Supplementary Fig. 5).

By the 6th dose of A-77636, animals displayed essentially zero contralateral rotational behavior. To achieve persistent receptor activation similar to A-77636, we administered six oral doses of the non-catechol agonist PF-2334 (10.8 mg/kg, Fig. 5d, Supplementary Fig. 6) following the same twice-daily schedule. The PF-2334 treatment led to rotational behavior over the course of doses 1 to 4 comparable to A-77636 (Supplementary Fig. 5). However, for doses 5 and 6, PF-2334 treated animals had significantly greater rotational behavior than A-77636 treated animals (Fig. 5d, *P* = 0.048, *P* = 0.022, respectively). Animals treated with PF-2334 were still displaying contralateral rotational behavior following the 6th dose. Furthermore, to confirm that the absence of rotational behavior was due to tachyphylaxis and not general fatigue, animals received a 7th subcutaneous dose consisting of the D2 agonist quinpirole (0.1 mg/kg) (Supplementary Fig. 5). During the PF-2334 treatment periods, administration of the D2 agonist induced a comparable number of contralateral rotations to the 6th non-catechol D1 dose, suggesting that the decrease in contralateral rotations over the time course of the 6 doses was at least partly due to behavioral fatigue (Supplementary Table 9). The persistent in vivo pharmacodynamic response of PF-2334 in primate and rodent models supports limited receptor desensitization by non-catechol agonists (Figs. 2 and 4), and suggests the potential for durable D1R agonism by non-catechols both in vitro and in vivo.

## Discussion

Selective agonism of D1 receptors has been a long-sought, but elusive strategy for the treatment of several neurologic and psychiatric diseases^13,21–23,54^. Here we have described a new class of non-catechol agonists with drug-like properties that potently and selectively activate D1Rs via a novel orthosteric site interaction. Although they show activity in established animal models associated with increased cAMP, this new class of D1R agonists does not induce significant desensitization and shows attenuated β-arrestin recruitment in vitro. Importantly, these D1 agonist compounds also show no or reduced tachyphylaxis in vivo—in contrast to known catechol agonists. These results provide a molecular basis for ligand-specific signaling bias and hold promise for the pursuit of D1R agonists as therapeutics.

All previously known D1R-selective agonists share a common pharmacophore which includes the dihydroxyphenyl catechol group. Although useful for uncovering D1R specific actions in experimental systems and establishing linkages to disease states, metabolic limitations rooted in the catechol structure have been the primary impediments to the investigation of D1R agonists as therapeutic agents^13,21–23^. Through tailored screening of a highly curated chemical file of almost 3 million compounds, we identified a single low potency non-catechol species and expanded upon it to yield a series of potent, selective D1R agonists. These novel compounds span a range of intrinsic D1R activities, and have highly favorable oral pharmacokinetic profiles. In addition to the anticipated utility of these compounds as tools to advance in vivo pharmacology and drug development efforts, these results define a novel molecular trigger for selective conformational changes needed for GPCR signaling via G-proteins and β-arrestins.

Ligands that exhibit functional selectivity to bias cytoplasmic signaling of GPCR′s toward either G proteins or β-arrestins have been described^47,49,55–58^. Yet, the structural basis by which specific ligand contacts favor GPCR conformations that direct cytoplasmic signaling toward or away from G proteins or β-arrestins remains controversial^45,56^. We have found that distinct molecular ligand-protein interactions within the extended orthosteric binding site of the D1R leads to conformational transit to G protein activation that either favors or disfavors subsequent β-arrestin binding. Specifically, our findings have shown that catechol hydrogen bonding with serines 198 and 202 on TM5 drives receptor activation that transitions into the β-arrestin bound state and produces canonical desensitization of the cAMP response. Simultaneous ionic interactions with TM5 serines and TM3 aspartate may enforce a long-lived and stable relative positioning of those helix segments which, in turn, transduce receptor conformations supporting recruitment of β-arrestin. Non-catechol agonists, however, engage the orthosteric binding site in a previously undescribed manner which includes important contacts with residues in ECL2. This activation mode drives potent G protein-mediated increases in cAMP, but does so with greatly reduced recruitment of β-arrestin or desensitization upon prolonged activation.

Although we cannot rule out that there may be additional, subtle structural or conformational contributions to the observed series-level bias, these results support a novel D1R binding state and provide a molecular basis for ligand-mediated β-arrestin signaling bias. Moreover, although recent structural studies have suggested that the direct hydrogen bonding of catechol hydroxyl moieties to TM5 serines is critical for agonism of β2AR^19^ and D1R^46,59,60^, we have shown that hydrogen bonding interactions with TM5 serines 198 and 202 is not a general requirement for acquisition of the D1R agonist-activated conformation. However, it remains plausible that there are multiple mechanisms by which synthetic D1R ligands can induce biased signaling. For example, some synthetic catechols may induce unique D1R conformations that may result in a functional bias^49^, and further studies of additional catechol and non-catechol pharmacophores are warranted. The structure of the D1R has not been determined; therefore an important extension of this work will be to define the precise nature of catechol and non-catechol agonist D1R binding via X-ray crystallography to more fully determine how ligand engagement with TM5 and ECL2 induces conformational changes in D1R transmembrane and cytoplasmic domains. Nonetheless, the finding of distinct orthosteric binding modes favoring G protein signaling independent of β-arrestin may provide a general basis for the design of functionally selective ligands at diverse GPCRs. Although additional studies are needed to increase our understanding regarding the functional impact of D1R-mediated β-arrestin signaling, specifically how arrestin contributes to in vivo drug tolerance, these findings suggest tachyphylaxis may be minimized by designing GPCR agonist ligands that have reduced arrestin engagement.

The series-level functional differentiation between catechol D1R agonists and non-catechol D1R agonists extended in vivo, where non-catechol D1R agonists show a durable in vivo pharmacological response without functional tachyphylaxis. The robust and sustained activity of orally-administered PF-2334 in both non-human primate eye blink response and the unilateral 6-OHDA lesioned rodent model of parkinsonism is consistent with the reduced β-arrestin recruitment and desensitization in vitro, and highlights the potential for compounds from this new class of non-catechol D1R selective ligands to address shortcomings with prior selective D1R ligands. The favorable oral pharmacokinetics and robust, sustained functional responses observed with non-catechol D1R agonists in vivo may enable testing of several long-standing therapeutic hypotheses for activating D1R in neurologic and psychiatric illnesses including Parkinson′s disease and schizophrenia.

## Methods

### cAMP assays

Human, rat, or monkey (*Macaca fasicularis)* dopamine D1/D5 receptor (D1R) agonist activity was measured using the Cisbio Dynamic 3′-5′-cyclic adenosine monophosphate (cAMP) homogeneous time-resolved fluorescence (HTRF) competitive immunoassay detection kit (Cisbio International 62AM4PEJ) according to the manufacturer's suggested protocol with minor amendments. For the D1 screening assay, including high throughput screening for non-catechol D1 agonists, we selected a stable hD1R-expressing clone, which was generated by transfecting hD1R into a HEK293 background line that we have maintained internally for a number of years. The clone was selected from more than a dozen other hD1R clones on the basis of performance in initial cAMP screening experiments, including cAMP assay hpe/zpe ratio and growth and stability characteristics. For all of the cAMP assays, the expression of the clone was confirmed by sequencing. Stable HEK293T cells expressing hD1R (wide type or mutant) were grown in high glucose DMEM (Invitrogen 11995-065), 10% fetal bovine serum dialyzed (Invitrogen 26400–044), 1 × MEM NEAA (Invitrogen 1140), 25 mM HEPES (Invitrogen 15630), 1 × penicillin/streptomycin (Invitrogen 15070-063) and 500 µg/mL genenticin (Invitrogen 10131-035) at 37 °C and 5% CO_2_. At 72–96 h post seeding, cells were rinsed with phosphate buffered saline and 0.25% trypsin-EDTA was added to dislodge the cells. Media was then added and cells were centrifuged and media removed. The cell pellets were resuspended in Cell Culture Freezing Medium (Invitrogen 12648-056) at a density of 40 million cells/ml. One ml aliquots of the cells were made in Cryo-vials and frozen at −80 °C for use in the hD1R HTRF cAMP assay. Stable HEK293 cells expressing hD5R (HD Bioscience) were grown in DMEM (Gibco 11965), 10% fetal bovine serum dialyzed (Sigma 058K0362), and 1× penicillin/streptomycin (Gibco 15070-063). Cells were frozen down at a density of 10 million cells/ml, then stored at −80 °C for use in the hD5R HTRF cAMP assay.

Frozen cells were quickly thawed, re-suspended in warm media and allowed to sit for 5 min prior to centrifugation (1000 rpm) at room temperature. Media was removed and the cell pellet was re-suspended in PBS containing 500 μM isobutylmethylxanthine (IBMX) to inhibit PDE activity. Using a Multidrop Combi (Thermo Scientific), 5 µL cells/well at a cell density of approximately 1000 cells/well were added to the assay plate (Greiner 784085) which contained 5 µl of test compound. To control for subtle cell density plating differences, each plate also contained positive controls of 5 µM dopamine (final concentration) and negative controls of 0.5% DMSO (final concentration). Cells and compounds were incubated at room temperature for 30 min. Working solutions of cAMP-D2 and anti-cAMP cryptate were prepared according to Cisbio instructions. Using the Multidrop Combi, 5 µL cAMP-D2 working solution was added to the assay plate containing the test compound and cells. Using the Multidrop Combi, 5 µL anti-cAMP-cryptate working solutions was added to assay plates containing test compound, cells, and cAMP-D2. Assay plates were incubated for 1 h at room temperature, then read using an Envision plate reader (Perkin Elmer) using Cisbio recommended settings. A time resolved and ratiometric emissions measurement (665 nm/620 nm) was obtained, which was then converted to cAMP concentrations using a standard curve. A cAMP standard curve was generated using cAMP stock solution provided in the Cisbio kit, which was then used to convert the raw ratio data to cAMP concentrations. EC_50_ values were determined using a logistic 4 parameter fit model to an 11 point concentration response curve with half-log increments. The percent efficacy for each curve was determined by the maximum asymptote of that fitted curve, and expressed as a percent of the maximum response produced by the positive controls (5 µM dopamine) on each plate. Reported values are the mean of results obtained across at least three independent experiments (*n* ≥ 3) each assayed in triplicate. The large file HTS screen protocol varied slightly from the subsequent SAR protocol in following areas: the HTS screen was run in 384 well plates containing 14 compounds/well and in the presence of an EC_10_ of dopamine present in all assays wells. Additionally, the ratiometric emissions measurement (665 nm/620 nm) was used to calculate % effect directly without the conversion to cAMP from the standard curve. Potential hits were de-convoluted and re-tested in the assay for confirmation at 20 µM compound concentration in a single compound/well format and without EC_10_ of Dopamine.

### Off target pharmacology profiling

FLIPR® calcium assay: Cells used in the assay were stably transfected with the receptor of interest (adrenergic alpha1a, dopamine 1, histamine 1, muscarinic 1, and serotonin 2b). Activation of the receptor by an agonist in this assay system results in an increase in intracellular calcium levels which is measured using a calcium specific dye. Cells were plated at 7500 cells per well (50 µl per well) in black walled clear bottomed 384-well plates 24 h prior to running the assay. Medium was removed from the plates and 80 µl of Hanks balanced salt solution (HBSS)/HEPES containing Calcium 5 dye (Molecular Devices; Cat # R8186) and probenecid (1.25 mM) was added to each well and the plate was returned to the incubator for 1 h to allow dye loading. Compound solution (10 µl) was added to each well by the FLIPR Tetra® instrument to measure agonist activity of the compound by measuring the change in fluorescence from baseline over a 60 s period (Ex 470–495 nM; Em 515–575 nM). Subsequently 10 µl of agonist (EC_80_ value) was added to each well by the FLIPR Tetra® instrument to evaluate antagonist activity, with the change in fluorescence from baseline being measured over a 60 s period.

Beta arrestin assay: The beta arrestin assay relies on enzyme fragment complementation with the respective stably transfected GPCR (adrenergic beta 2, cannabinoid 1 and mu opioid) being tagged with an inactive portion of the enzyme β-galactosidase and a cotransfected β-arrestin that is tagged with the complementary portion of β-galactosidase. Recruitment of β-arrestin to the GPCR, results in a functional enzyme that generates a chemiluminescent signal when substrate is added. Cells were plated at 5000 cells per well (40 µl per well) in black walled clear bottomed 384-well plates 24 h prior to running the assay. Medium was removed from the plates. For agonist studies 15 µl of HBSS/HEPES containing compound was added to the cells and the plate was incubated at room temperature for 90 min. For antagonist studies 15 µl of HBSS/HEPES containing compound was added to the cells and was incubated for 15 min prior to the addition of 15 µl of an EC_80_ concentration of agonist. The plate was subsequently incubated at room temperature for 90 min. Both assays were terminated by addition of 15 µl of a Beta-Glo® solution (Promega). Following an additional 30 min incubation the luminescence of each well was measured to determine the level of receptor activation.

Amine transporter assay: The amine transporter assay measures the ability of compounds to inhibit the activity of the norepinephrine (NET) dopamine (DAT) or serotonin (SERT) transporters by measuring the real time uptake of a dye labeled amine. HBSS/HEPES containing compound (5 µl) was added to the wells of black walled clear bottomed 384-well plate. Transporter dye (25 µl) (Molecular Devices; Cat # R8174) was added to each well. Finally 15,000 cells (20 µl) stably expressing the amine transporter of interest were added to each well and the plate is incubated at 37 °C for 30 min (DAT) or 60 min (NET and SERT). The plate is transferred to the FLIPR Tetra® instrument and the fluorescence of each well was measured (Ex 470–495 nM; Em 515–575 nM). The level of fluorescence measured directly relates to the level of uptake of the dye labelled amine, with a reduction in levels being related to an inhibition of the respective transporter.

Ion channel assays: L-type calcium channel activity was measured in the H9C2 cell line. Cells were plated at 5000 cells per well (50 µl per well) in black walled clear bottomed 384-well plates 72 h prior to running the assay. Medium was removed from the plates and 20 µl of HBSS/HEPES containing calcium 5 dye (Molecular Devices; Cat # R8186) was added to each well and the plate was returned to the incubator for 1 h to allow dye loading. Compound solution (5 µl) was added to each well by the FLIPR Tetra® instrument to measure agonist activity of the compound by measuring the change in fluorescence from baseline over a 60 s period (Ex 470–495 nM; Em 515–575 nM). Subsequently 25 µl of a high KCl buffer (in mM: KCl, 140; MgCl_2_, 1; HEPES, 20; Glucose, 10, CaCl_2_, 10) was added to each well by the FLIPR Tetra® instrument to evaluate antagonist activity, with the change in fluorescence from baseline being measured over a 60 s period.

Sodium channel (Nav1.5) activity was assessed using a FLIPR® based membrane potential assay using cells that stably express the Nav1.5 sodium channel subtype. Cells were plated at 7500 cells per well (50 µl per well) in black walled clear bottomed 384-well plates 24 h prior to running the assay. Medium was removed from the plates and 80 µl of (HBSS)/HEPES containing membrane potential dye (Molecular Devices; Cat # R8123) was added to each well and the plate was returned to the incubator for 1 h to allow dye loading. Compound solution (10 µl) was added to each well by the FLIPR Tetra® instrument to measure agonist activity of the compound by measuring the change in fluorescence from baseline over a 60 s period (Ex 510–545 nM; Em 565–625 nM). Subsequently 10 µl of agonist (EC_80_ value) was added to each well by the FLIPR Tetra® instrument to evaluate antagonist activity, with the change in fluorescence from baseline being measured over a 60 s period.

PDE assays: The PDE assays measures the conversion of 3′, 5′-[^3^H] cAMP to 5′-[^3^H] AMP (for PDE 3A1 and 4D3) or 3′, 5′-[^3^H] cGMP to 5′-[^3^H] GMP (for PDE 5A1) by the relevant PDE enzyme subtype. Yttrium silicate (YSi) scintillation proximity (SPA) beads bind selectively to 5′-[^3^H] AMP or 5′-[^3^H] GMP, hence the magnitude of radioactive counts is directly related to PDE enzymatic activity. The assay was performed in white walled opaque bottom 384-well plates. One microliter of compound in dimethyl sulfoxide was added to each well. Enzyme solution was then added to each well in buffer (in mM: Trizma, 50 (pH7.5); MgCl_2_, 1.3 mM). Subsequently, 20 µl of 3′,5′-[^3^H] cGMP (125 nM) or 20 µL of 3′,5′-[^3^H] cAMP (50 nM) was added to each well to start the reaction and the plate was incubated for 30 min at 25 °C. The reaction was terminated by the addition of 20 µl of PDE YSi SPA beads (Perkin Elmer). Following an additional 8 h incubation period the plates were read on a MicroBeta radioactive plate counter (Perkin Elmer) to determine radioactive counts per well.

In vitro data analysis: Agonist/antagonist curves were plotted from individual experiments, and EC_50_/IC_50_ values were determined using a four parameter logistic fit. EC_50_ is defined as the concentration of the test article that produced a response that was equal to 50% of the maximal system response. IC_50_ is defined as the concentration of the test article that produced a 50% inhibition of a maximal response.

An apparent Kb value for antagonist activity was calculated using the following equation (Eq. 1):

1 $$\mathrm {Apparent}\,\mathrm {Kb} = \mathrm {IC}_{50}/\left( {1 + \left( {\left[ A \right]/\mathrm {Agonist}\,\mathrm {EC}_{50}} \right)} \right.$$

where the Kb value is the dissociation constant of antagonist for the receptor, IC_50_ is the response produced by the test article in the presence of [*A*], the concentration of agonist used in the assay. Agonist EC_50_ is the EC_50_ value of the reference agonist used in the assay when tested alone.

### Radioligand binding assays

Binding assays were performed using stably expressed human D1R LTK, HEK293 rat D1R (rD1R), CHO human D2R (hD2R), CHO human D3R, and Millipore CHEM-1 human D5R (HTS129M) cell lines or primary striatal tissue from cynomolgus monkey brain. The background LTK, HEK293, and CHO cell lines have been maintained at Pfizer for use in screening assays for a many years and expression was confirmed by sequencing. To determine basic assay parameters, ligand concentrations were determined from saturation binding studies where the *K*_d_ using [^3^H]-SCH23390 (Perkin Elmer NET930001MC) was found to be 1.3 nM for hD1R, 0.5 nM for rD1R, 1.7 nM for monkey striatal tissue and 4.2 nM for D5R. For D2R binding, a *K*_d_ of 1.6 nM using [^3^H]-spiperone (Perkin Elmer NET1187250UC) was determined and a *K*_d_ of 1.4 nM for hD3R using [^3^H]−7-OH-DPAT. From tissue concentration curve studies the optimal amount of tissue was determined to be 2.4 mg/ml for hD1R and hD3R, 1.8 g/ml for rD1R, 9.0 mg/ml for monkey striatum and 4 mg/ml for D2R and D5R per 96 well plate. These ligand and tissue concentrations were used in time course studies to determine linearity and equilibrium conditions for binding. Binding reached equilibrium with the specified amount of tissue by 30 min at 37 °C for hD1R, rD1R, mD1R, 20 min at 37 °C for D2R and D3R and 60 min at room temperature for D5R. From these parameters, *K*_i_ values were determined by homogenizing the specified amount of tissue for each receptor in 50 mM Tris (pH 7.4 at 4 °C) containing 2.0 mM MgCl_2_, and spun in a centrifuge at 40,000 × g for 10 min. The pellet was resuspended in either the D1/D5 assay buffer (50 mM Tris (pH 7.4 at RT) containing 4 mM MgSO_4_ and 0.5 mM EDTA), the D2 assay buffer (50 mM Tris (pH 7.4 at 37 °C), 100 mM NaCl and 1 mM MgCl_2_) or the D3 assay buffer (50 mM Tris containing 120 mM NaCl, 5 mM MgCl_2_, 5 mM KC, and 2 mM CaCl_2_ at pH 7.4 at 37 °C. Incubations were initiated by the addition of 200 µl of tissue to 96-well plates containing test drugs (2.5 µl) and 0.5 nM [^3^H]-SCH23390 for hD1R, rD1R, and mD1R, 1.0 nM of [^3^H]-SCH23390 for D5R, 2 nM [^3^H]-spiperone for D2R or 1.5 nM of [^3^H]-7-OH-DPAT for D3R (all 50 µl) for a final assay volume of 250 μl. Non-specific binding was determined by radioligand binding in the presence of a saturating concentration of (+)-butaclamol (10 μM), for both D1R and D5R, fluphenazine (10 μM) for D5R and haloperidol for D2R and D3R. After the specified incubation period at 37 °C, assay samples were rapidly filtered through Unifilter-96 GF/B PEI-coated filter plates and rinsed with ice-cold 50 mM Tris buffer (pH 7.4 at 4 °C). Membrane radioligand levels were determined by liquid scintillation counting of the filter plates in 50 µl Ecolume. The IC_50_ value (concentration at which 50% inhibition of specific binding occurs) was calculated by linear regression of the concentration-response data (10 concentrations at half-log increments) in Activity Base (IDBS). *K*_i_ values were calculated according to the Cheng-Prusoff equation. Reported values are the mean of results obtained across at least three independent experiments (*n* ≥ 3) each assayed in triplicate.

### Assessing competitive interaction between SCH23390 and PF compounds

Radioligand: binding assays were performed using LTK cells expressing human dopamine D1R. Saturation studies were carried out in the presence and absence of PF compound to determine how PF compounds interacted with the receptor (e.g., competitive, non-competitive manner, etc.). Briefly, frozen D1R cell pellet (2.4 mg/mL) was homogenized with a Brinkman Polytron in 50 mM Tris (pH 7.4 at 4 °C) containing 2.0 mM MgCl_2_, and spun in a centrifuge at 40,000 × g for 10 min. The pellet was re-suspended in D1 assay buffer (50 mM Tris (pH 7.4 at RT) containing 4 mM MgSO_4_ and 0.5 mM EDTA). Incubations were initiated by the addition of 200 µl of tissue homogenate to 96-well plates containing varying concentrations of [^3^H]-SCH23390 (Perkin Elmer NET93000; eight concentrations ranging from 0.2 nM to 15 nM) in presence or absence of PF compound in a final assay volume of 250 μl. Total binding was determined by DMSO and non-specific binding was determined by radioligand binding in the presence of a saturating concentration of (+)-butaclamol (10 μM). After a 30 minute incubation at 37 °C, assay samples were rapidly filtered through Unifilter-96 GF/B PEI-coated filter plates and rinsed with ice-cold 50 mM Tris buffer (pH 7.4 at 4 °C). Membrane bound radioligand levels were determined by liquid scintillation counting of the filter plates in 50 µl Ecolume. Saturation binding data was analyzed using GraphPad Prism (6.0) for each condition (total, in presence of PF) and yielded *K*_d_ and *B*max values for each condition.

### Analysis of data from competition binding studies

A hierarchical Bayesian model was used to estimate the parameters of the Michaelis-Menten model by group (e.g., low concentration, high concentration, and total) and date within group. Three chains were used with a total of 50,000 MCMC iterations with a burn-in of 25,000 iterations. The chains were evaluated for convergence. Using the Bayesian analysis, the 95% credible intervals were be computed with separate *B*max and *K*_d_ parameters per group (i.e., total, 3.2 nM, and 10.0 nM). A separate analysis was conducted for each compound. There were 216 data points measured at nine concentrations of 3H-SCH23390 each on three separate days for each compound. No apparent outliers were removed from the analysis. The estimated parameters were for *B*max and *K*_d_ for PF-6142, PF-8871, and PF-1437 along with confidence bounds are given in Supplementary Table 5. For PF-6142, the asymptote for the 3.2 nM dose group is higher than the total, and the difference is not larger than the experimental noise and should not be considered a substantial difference. For the higher dose, the asymptote is significantly less than the results from the total group. This analysis suggests that PF-06256142 interacts in a manner consistent with competitive inhibition at 3.2 nM. For PF-8871, both asymptotes for the 3.2 and 10.0 nM dose groups are smaller than the total and the differences are larger than the experimental noise. This analysis suggests that PF-06258871 interacts in a manner consistent with mixed competitive/non-competitive inhibition at 3.2 nM and 10.0 nM. For PF-1437, both asymptotes for the 3.2 and 10.0 nM dose groups are smaller than the total and the differences are larger than the experimental noise. This analysis suggests that PF-06481437 interacts in a manner consistent with mixed competitive /non-competitive inhibition at 3.2 and 10.0 nM.

### Rat pharmacokinetic studies

Rat pharmacokinetic studies were conducted at BioDuro, Pharmaceutical Product Development, Inc. (Shanghai, China). Wistar Hannover rats (227–293 gram weight) were obtained from Vital River Laboratories (China). All animal studies were conducted in accordance with the Pfizer Institutional Animal Care and Use Committee (Groton, CT), using protocols approved by Pfizer PDM′s Global BA Committee (2009). Jugular vein-cannulated male Wistar Hanover rats were maintained on a 12 h light/dark cycle for a minimum of three days in a temperature-controlled and humidity-controlled environment with free access to food and water. Individual animals (*n* = 2 or 3 per dose/route) were administered a single dose of compound by either intravenous injection via tail vein or oral gavage based on body weight. SKF-38393 intravenous (1 mg/kg) and oral (5 mg/kg) doses were formulated in distilled water. The PF-6142 intravenous dose (1 mg/kg) was formulated in saline acidified with three molar equivalents of 1 N hydrochloric acid and the PF-6142 oral dose (5 mg/kg) was formulated in 0.5% methylcellulose. The PF-2334 intravenous dose (0.1 mg/kg) was formulated in 5/5/90 (DMSO/cremophor/saline) and the oral dose (3 mg/kg) was formulated in 0.5% methylcellulose. Formulations were agitated until all of the test article was dissolved. For both SKF-38393 and PF-6142, the intravenous dose was administered at a volume of 2 ml/kg for and the oral dose was administered at 5 ml/kg. For PF-2334, the intravenous dose was administered at 1 ml/kg and the oral dose was administered at 10 ml/kg. From each rat, serial blood samples were collected via jugular vein cannula prior to dose administration and at the following time points post-dose: 0.033 (i.v. only), 0.083, 0.25, 0.5, 1, 2, 4, 7, and 24 h. For PF-6142 and PF-2334, blood samples were placed into tubes containing EDTA. For SKF-38393, blood samples were collected in tubes containing ascorbic acid (final concentration 40 mM) and EDTA. All blood samples were placed on wet ice until centrifugation. Following centrifugation, the plasma was transferred to polypropylene tubes and stored frozen at either −20 °C to −80 °C until analysis. Urine was collected from all intravenous-dosed rats into collection tubes from the collection intervals of 0–7 h and 7–24 h post-dose. For SKF-38393, ascorbic acid was added at the end of each urine collection interval (40 mM final concentration). All urine samples were stored at −80 °C until analysis. All samples were processed for quantitation of compound concentrations using protein precipitation methodology followed by analysis using liquid chromatography-tandem mass spectrometry (LC-MS/MS). The preparation of standard curves for quantitation of SKF-38393 included ascorbic acid (40 mM final concentration). The pharmacokinetic parameters (including those in Supplementary Table 3) were computed by non-compartmental analysis^61^ using Watson Bioanalytical LIMS version 7.4 (Thermo Fischer Scientific, CA).

Nonspecific binding studies: Equilibrium dialysis and standard procedures were used for determination of the unbound fraction of PF-2334 and A-77636 in monkey plasma (*f*_u,p_)^62^ and rat brain homogenate (*f*_u,b_)^63^. For A-77636, each matrix contained ascorbic acid (40 mM final concentration) to ensure compound stability. The mean *f*_u,p_ and *f*_u,b_ values for PF-2334 were 0.040 and 0.021 and the mean *f*_u,p_ and *f*_u,b_ values A-77636 are 0.044 and 0.0012.

Quantitation of PF-2334, SKF-38393, and A-77636 in biological matrices: All samples (rat and monkey plasma and nonspecific binding study samples) were processed for quantitation of compound concentrations using protein precipitation methodology followed by analysis using LC-MS/MS. The preparation of standard curves for quantitation of SKF-38393 and A-77636 included the addition of ascorbic acid (40 mM final concentration) to ensure compound stability.

### D1R desensitization studies

Striatal neurons were chosen for the desensitization studies because they express endogenous D1 receptors and are a physiologically relevant tissue for examining neurotransmitter receptor desensitization in vitro. Primary rat striatal neurons were obtained from embryonic day 18 (E18) male and female Sprague-Dawley rats by standard neuronal isolation procedures, Embryos were isolated from pregnant rats, 18 days after timed matings, and placed into Hanks buffered saline solution (HBSS) supplemented with 100 U/mL penicillin/streptomycin, 10 mM MgCl, 2 mM L-Glutamine and 7 mM HEPES. Both hemispheres of the striatum were microdissected from the embryonic rat brains and placed into HBSS. All isolated tissue was combined and washed 1× with HBSS, followed by trypsinization using a 0.05% trypsin-EDTA solution for 15 min at 37 °C. Proteolysis was stopped by adding an equal volume of complete MEM medium (MEM Earle′s salts 100U/ml penicillin/streptomycin, 2mM L-Glutamine and 10% Fetal Bovine Serum (Hylcone, defined and heat inactivated), followed by tissue trituration by passing the tissues 10–15× through a pasteur pipette until a single cell suspension was obtained. The cell suspension was added to the top of 5 ml complete MEM media with 1 ml of 100% FBS serum at bottom of a 15 ml tube and centrifuged at 1450 rpm for 5 min. The supernatant was obtained and diluted 10–20× in complete MEM and cell density determined. Cells were plated at a density of 35,000 cells/well in poly-ornithine/laminin coated 96 well plates (Biocoat, BD Falcon) and cultured in Neurobasal media supplemented with B27, 1× Glutamax, and penicillin/streptomycin (100 U/mL) (all from Invitrogen) at 37 °C in 5% CO_2_ for 14–16 days prior to assay. To assess D1R desensitization, neurons in wells were pretreated for 120 min with 0.1% DMSO (vehicle) or 10 μM of test compounds dissolved in serum free Neurobasal media. After the pretreatment, cells were washed twice at 5 min intervals with 250 μl/well fresh Neurobasal media. The ability of D1Rs to still signal was then examined by treating cells for 30 min with 1 μM SKF-81297, a catechol D1 selective full agonist, in the presence of 500 μM IBMX. The concentration of cAMP accumulated in each well was determined using the Cisbio HTRF cAMP dynamic range assay kit (Cisbio) according to the manufacturers suggested protocol. The concentration of cAMP (nM) from treated wells was interpolated from a cAMP standard curve by non-linear regression least squares analysis using Graphpad Prism 5.02. The mean ± standard error of the cAMP concentrations were calculated from results obtained across three independent experiments (*n* = 3) each assayed in triplicate. The percent desensitization was calculated as the percent decrease in cAMP relative to vehicle control. Statistical differences were compared by a one-way ANOVA with Dunnett′s post-test analysis using Graphpad Prism 5.02. Longer treatment periods of striatal neurons for up to 12 h with saturating (10 μM) concentrations of selected non-catechols D1R agonists were examined. In line with data reported herein, these D1R agonists failed to significantly induce desensitization of cAMP; however, under the reported assay conditions, cell health and particularly total cAMP levels were notably impacted at the longer incubation times (even for vehicle treatment), obscuring interpretation (data not shown).

### β-arrestin membrane recruitment assays and TIRF microscopy

For all studies of β-arrestin, a stable U2OS cell line co-expressing human dopamine D1(D1A) receptors and human β-arrestin2-green fluorescent fusion protein (GFP) was used. This stable U2OS cell line was generated, obtained and licensed from Professor Marc G. Caron, Duke University, Durham, NC, USA. The cell line provides a fluorescent biosensor of β-arrestin2-GFP that can be used to assess GPCR signaling and GPCR-mediated β-arrestin membrane recruitment using imaging-based methods such as fluorescence microscopy (US Patents 7,572,888 and 7,138,240)^64^; the D1R/arrestin-GFP stable U2OS cell line and this technology is currently marketed as the Transfluor Assay (Molecular Devices, USA). The U2OS cells were cultured under antibiotic selection in DMEM (Invitrogen) containing 25 mM glucose and 4 mM L-glutamine supplemented with 10% dialyzed fetal bovine serum, 200 mg/mL Geneticin, 100 mg/mL Zeocin, and 100 U/ml penicillin/streptomycin (all from Invitrogen) and incubated at 37 °C in 5% CO_2_. Cells from passage four through ten were used in these experiments. Cells were grown in 35 mm glass bottomed imaging dishes (Mattek Corp). Cells were incubated for 1 h in serum free media (SFM) and subsequently treated for 10 min at 37 °C with 0.01% DMSO (control) or 1 μM of all test compounds dissolved in SFM followed by immediate fixation on ice with a 4% paraformaldehyde/1× phosphate buffered saline solution.

TIRFM was used to visualize proteins at the plasma membrane of cells such as D1 receptors and recruited β-arrestin-GFP^65^. All images were captured using a Zeiss PS.1 Elyra Superresolution fluorescence microscope equipped with TIRF module. Images of cells were obtained using TIRF and a 100× oil immersion objective and dedicated 488 nm excitation laser. Optimal exposure time and laser power was determined using dopamine-treated cells, which exhibited maximal β-arrestin-GFP membrane signal and identical acquisition parameters were used for all cells and conditions (Extended Data Fig. 1 and Supplementary Movies 1–4 contain additional images and views).

### Image analysis of TIRF microscopy

Custom image analysis scripts were developed to automatically identify and quantify β-arrestin membrane recruitment. We employed standard methods of image processing based on grayscale morphology^66^. First 16bit images were transformed to 8bit range via square root transformation to improve identification of punctate structure of varying intensity. Images were smoothed with small Gaussian kernel to eliminate noise (*h* = 2px, *σ* = 1). At the next step images were transformed via tophat operation with large kernel (radius 30px) to eliminate non-uniformity of the background. Second tophat transform with small kernel (radius 5px) was used to enhance signal and eliminate local intensity variation around puncta. This image was then processed to find locations of puncta intensity peaks via non-maximal suppression filter with 3px radius of region considered in non-maximal suppression, and threshold 20. Finally the boundaries of puncta were identified using marker controlled watershed operation with peaks serving as foreground markers. After having identified each puncta, we used 8bit transformed but otherwise non-processed images to quantify intensity and area of each region. We focused on two endpoints, number of puncta per cell and integrated intensity of puncta per cell (sum of the products of average intensity and area of each puncta within a cell), both adjusted for cell area as described below. The boundaries of the cells were identified via Otsu's method^67^, followed by hole filling and identification of the largest closely connected component. The area of the foreground was used as normalization factor to account for occasional differences in cell areas in the field of view. Specifically all foreground areas across experiment were pooled and divided by the median of the areas to obtain normalization factor for each image. This factor was then used for adjustment for number of puncta and integrated intensity of puncta, which scale with the number of cells. Puncta outside of cell boundaries were filtered out.

The above-mentioned parameters were tuned to achieve qualitative agreement between three independent raters, as well as with published segmentation results^68^. Results of segmentation were saved as overlays on the images for downstream quality control and inspected by independent expert. The choice of parameters was made to provide sufficient sensitivity and to enable post-hoc gating of puncta based on area and intensity parameters. We tested that more stringent threshold on puncta intensity yielded statistically equivalent findings for group differences.

The results were aggregated over batches of cells recorded at the same time, with three or more batches per condition, approximately 20 cells per well. The statistical significance was tested via one-way ANOVA with treatment as a factor. To address the problem of severe inequality of variances across the treatment groups assessed via Bartlett′s test, we used square root transformation. All statistical results for the number of puncta per cell and integrated intensity of puncta per cell are presented for transformed variables. Significant ANOVA findings were followed with post-hoc comparisons with Dunnett′s procedure in case of comparisons with the control group or with false discover rate adjustment^69^ otherwise. All statistical tests were performed two-tailed at a 5% level of significance. Image processing algorithms were implemented in MATLAB (Mathworks Inc., Natick, MA, R2010b), while post-processing and statistical analysis was performed in R^70^.

### D1R mutagenesis and functional characterization

A wildtype 3xHA-human6 D1R expression construct (in pcDNA3.1+) was obtained from Missouri S&T cDNA Resource Center. Several mutations were created using mutagenesis methods (e.g., Stratagene Quick Change Mutagenesis Kit). All mutations were confirmed via sequencing. Mutants were designed based upon a computational homology model of the D1R. Relative 3xHA-hD1 mutant expression levels were normalized to wild type hD1 levels by immunoblot analysis. Soluble RIPA lysates of transiently transfected HEK293F cells were prepared by lysing cells at 4 °C for 30 min in RIPA Buffer (Sigma) with protease and phosphatase inhibitors (Pierce). Equivalent amounts of total soluble RIPA lysates (determined by BCA total protein assay, Pierce) were run on SDS-PAGE, transferred to nitrocellulose and probed with anti-HA as well as anti-GAPDH antibodies (Sigma). Total mutant hD1 HA immunoreactivity was quantitated versus GAPDH immunoreactivity (HA/GAPDH) and then normalized to wild type 3xHA-hD1 (HA/GAPDH) using LiCor/Odyssey software. Based on HA/GAPDH ratios compared to wildtype, the relative amount of cell paste (for binding studies) or cell numbers/well was adjusted for each mutants' expression level for functional cAMP assays. Wild type and mutant(s) expressing HEK293 cells were generated (for both binding and cAMP assays) via transient transfection (48 h) in Freestyle HEK 293 F cells (Invitrogen) and agonist-induced cAMP production determined using the Cisbio HTRF cAMP assay as described in the methods section for cAMP assays. To compare the relative effects of mutations on the signaling of all catechol or non-catechol agonists (Fig. 3g, f), agonist responses for the series of ligands were combined and normalized to the wildtype receptor responses measured in the same experiment (Δpotency = EC_50mut_/EC_50wt)_. Statistical significance for structural series-level Δpotency was determined for each mutant by One-way ANOVA with Holm Post-hoc analysis. Figure 4f expresses mean ± standard error Δpotency calculations for dopamine and PF-1437 (*n* ≥ 3), and *n* = 1 data for PF-8871. Supplementary Table 6 contains individual compound-level Δpotency calculations for each experiment conducted.

### Homology docking models

The amino acid sequences of the human D1R were obtained from the web server UniProtKB (http://www.uniprot.org) and were aligned with the β2-adrenergic receptor (pdb code: 3P0G)^66^ using the MOE sequence alignment tool (Chemical Computing Group). The homology model was built using the 3P0G crystal structure and the MOE homology model builder, and energy minimized using the CHARMM force field. Neither the N-terminus or C-terminus was modeled due to those regions not being visible in the template. The sequence begins at R18 and ends at G346. Poses of catechol standards shown in Fig. 3c were generated using the in-house docking program AGDOCK^71^ AGDOCK uses a variation of the AMBER force field that utilizes a soft-core AMBER function a piecewise linear energy function, with the intent of avoiding rapidly changing/high energy landscapes, and thus sampling more conformational space. As reported previously^20^, docking experiments were used to approximate the placement of catechol-amine structures into the orthosteric site of the D1r homology model. “Standard” ligands were docked in the protonated form. The best scoring ligand–protein structures were then subject to a minimization in which the protein is allowed to be flexible within a 6 Å shell (constrained with a restraining force of 500 kcal/Å^2^). The Schrodinger Batchmin (v14) with AMBER Force Field was used for this step, with a 12 Å non-bonded cutoff with a distance-based dielectric solvation model (eps = 4)^72^. In previous work, we have reported the structure of SKF-38393, as well as additional examples of catechol amines, docked into our D1r homology model^20^. Similar to previously outlined models^46^, the orientation of this ligand is driven by the amine-D1033.32 ionic interaction and the polar interface of the catechol with the TM5 S1985.42 and S2025.46 and possibly S1995.43 and N2926.55.

PF-compounds are reported here for the first time. Relative to catechol standards, the docking of the PFE seriers furyl-piperidines is more challenging, as neither the basic center of catechol is available to anchor the ligand in any obvious manner. Again, using our in-house docking program^71^ and the minimization scheme outlined above resulted in the poses shown in Figs. 3d, 4c, d. Additional views of these poses are shown in Supplementary Movies 5, 6, 8, and 9. These were then used as a starting point for hypothesis generation for compound design. One design highlighted here takes the pose from Fig. 4c on the left (with the furyl near the TM5 serine bundle) and substitutes the catechol off of the ether, in place of the furyl-pyridine (Fig. 4d). This merged pharmacophore compound was found to be active in both binding and functional assays. A similar design was also proposed for the pose on the right via placement of the catechol moiety onto the pyrimidine. Those analogues were found to be inactive and, based on this result and additional SAR (to be published), we have arrived at the pose in Fig. 4d in the main text as our proposed binding model for compounds in this series. Atomic distances and angles between selected ligands and the closest D1R atom are presented in Supplementary Table 4.

### Docking and molecular dynamics simulations

Initial coordinates and system setup: Two D1 receptor homology model (D1r-hm) simulations were initiated using the coordinates of the PF-8871 and PF-1437 docked into the D1R model^20^ and the results are presented in Supplementary Movies 7, 10, and 11. As before, only the D1R model was used in the simulations and no g-protein or nano body was used to stabilize the model receptor in an active state. As it is known that active GPCR structures deactivate during MD simulations^72^, only relatively short simulations were attempted and the system was monitored to insure deactivation does not take place.

Simulation details: The D1r-hm was embedded in a hydrated pre-equilibriated lipid bilayer (POPC) in all simulations; all atoms were represented explicitly. Hydrogen atoms were added and residue titration was accomplished using the protein-prep wizard in Maestro (Schrodinger, LLC). In total the systems contained 78 lipid molecules, 6356 water molecules, 10 chloride ions in addition to the ligand and protein model. The OPLS3 force field was used with the TIP3P water model. All simulations were performed using the DESMOND simulation package under periodic boundary conditions in a rectangular box at 300 K with Berendsen thermostat and coupling time constant of 1.0 ps. All systems were first subject to a temperature-ramping scheme including 5–10 ns of un-restrained equilibriation, followed by 40–50 ns production runs and a time step of 2 fs. All simulations were carried out on Dell Poweredge C6220 servers with NVIDIA Tesla M2075 GPUs. MD Quality Assesment. Trajectory snapshots were saved every 48 ps during production simulations. The Desmond Simulation Interaction Diagram plots are shown in Supplementary Figures 7 and 8. For both simulations, the RMSD for all atoms in the protein is in the 2–3 range and some drift is observed for both simulations. Simulations of the D1 homology model were all performed without a complexing partner (g-protein or T4-lysozyme), and are, based on previous simulations of active β2aR^47,72^, non-equilbrium simulations. As such, expectations are that common measures of equilibration will show some drift. Neither ligands drift in the simulation compared to their initial positioning. Protein–ligand contacts measured over the course of the simulation agree with those presented in the docking model from the text, and reflect the reported mutation data.

Using the Bio3D package, additional RMSD plots are reported in Supplementary Fig. 9. Here, the top plot shows the RMSD for all alpha-carbon atoms, and the bottom plots are all protein atoms, but with the residue selection restricted to the trans-membrane residues (as annotated by unitprot). Both simulations show reduction in the RMSD for transmembrane atoms, suggesting that a large contribution to RMSD is in the loop region. Some drift, however, is still observed. This may be due to a number of factors: latent inaccuracies of the homology model (loop placement being the main concern), improper membrane imbedding (although we observe no solvent leaking), or deactivation of the receptor model (non-equilibrium simulation). Since the primary goal of the simulation is to access ligand placement, this latter factor is a concern and has been previously observed in long-time simulations of the active b2ar receptor. Dror et al. ^72^ have monitored receptor deactivation using the NPxxY (Asn322-Tyr326 in D1r) region rmsd to frame 0 (start of the averaging) and helix 6-helix 3 distance as measured from the Ca atoms of Arg121 (helix 3) to Leu 271 (helix 6). Using these metrics (Supplementary Fig. 10), we observe no evidence of receptor deactivation, and the helix-6-helix-3 distance is in the 13–16 ang. range, consistent with those observed for β2ar simulations^72^.

### Monkey EBRs

Increase in spontaneous EBR has been previously shown with D1 agonists and partial agonists^51^. To confirm the specificity of the response in our particular assay set up for D1 activation, we verified that the D1 receptor antagonist SCH-39166 fully blocks the increased eye blink response of non-catechol full D1 agonist that is a close structural analog of the compounds in this paper (data not shown). All procedures involving animals were conducted with approval of the Pfizer Institutional Animal Care and Use Committee (Groton, CT) and compliant with the regulations and standards of the Animal Welfare Act (9CFR2, 9CFR3). Three male Cynomolgus macaques (*Macaca fasicularis*) of Mauritian origin, 7–10 years old, SPF for CHV-1, SIV, STLV1 and SRV 1, 2, 5 were used. Animals were exposed to 12 h light/dark cycle (lights on 6:00 AM – 6:00 PM). Prior to in-life study, animals were found to be free of any significant ophthalmic lesions by a veterinarian and acclimated to chair restraint. The compound PF-2334 and vehicle were administered orally and A77636 was dosed subcutaneously. EBRS were recorded from chaired animals for 2 min at 10 min intervals by a trained observer through direct visual observation who was blinded to experimental conditions. Animals were chaired at approximately 7:30 AM. After vehicle or compound administration, eye blinks were recorded during the following intervals: from 10 to 50 min (0–1 h), 130 to 170 min (2–3 h), 310 to 350 min (5–6 h). Animals were returned to their home cages between two consecutive sessions and after the final recording of the day. Blood samples (1 ml) were collected from the femoral vein at 1 and 5 h after the first dose to quantitate plasma compound levels. PF-2334 was suspended in 0.5% methylcellulose acidified with 1 M equivalent (Me) HCl within 24 h of administration, PF-2334 formulations were kept at room temperature, and stirred continuously prior to and during administration to ensure homogeneity. A77636 was dissolved in 0.9% sterile saline, formulated fresh prior to administration, and given once per day for 3 days. PF-2334 was administered twice daily, (0.6 mg/kg at 8:00 AM and 0.3 mg/kg at 4:00 PM), from day 1 through day 3) to mimic the brain D1R receptor occupancy of A77636 administered subcutaneously (1 mg/kg at 8:00 AM from day 1 through day 3). Vehicle control was administered on day 0.

Monkey EBR study pharmacokinetic analysis: In the monkey EBR study for PF-2334, blood samples (1 mL) were collected from the femoral vein and placed into tubes containing EDTA at 1 and 5 h after the first daily dose. For A-77636, the blood samples (1 mL) were collected from the femoral vein and placed into tubes containing ascorbic acid (final concentration 40 mM) and EDTA at 1 and 6 h after the first daily dose. For both compounds, blood collections occurred on day 1, day 2, and day 3. All blood samples were placed on wet ice until centrifugation. Following centrifugation, the plasma was transferred to polypropylene tubes and stored frozen at –20 °C to –80 °C until analysis. The plasma concentrations of PF-2334 and A-77636 are located in Supplementary Table 7 and Supplementary Table 8, respectively.

Pharmacokinetic modeling and simulations for monkey EBR study dose selection and estimation of central receptor occupancy: Pharmacokinetic and neuropharmacokinetic studies were conducted in monkeys with PF-2334 and in monkeys with A-77636 to determine the plasma and brain concentration-time profiles (data not shown). As the compounds differ in regard to plasma pharmacokinetic properties and brain penetration (both rate and extent), the data for each compound was modeled and the resulting pharmacokinetic parameters were used to simulate doses for the monkey EBR study. The objective of the modeling was to select the appropriate dose(s) and dose regimen for each compound, normalizing the exposure coverage to ensure a similar duration and extent of projected central receptor occupancy. Normalizing the compounds on the basis on projected central receptor occupancy takes into account the differences in the compounds in relation to unbound brain concentrations (*C*_b,u_) and D1 binding Ki. The projected central receptor occupancy of each compound was estimated through the use of *C*_b,u_ and the human binding Ki at the D1 receptor through use of the following equation (Eq. 2):

2 $$ {\mathrm{RO}}\left( \% \right) = \left[ {C_{\mathrm{{b,u}}}/\left( {C_{\mathrm{{b,u}}} + {\mathrm{K}_{\rm i}}} \right)} \right] \times 100$$

The projected central receptor occupancy profiles of PF-2334 and A-77636 from the doses administered in the monkey EBR study are illustrated in Supplementary Fig. 4. The plasma concentration data collected during the EBR study for PF-2334 and A-77636 were used as confirmation that the targeted peripheral exposures were achieved during the study conduct.

### Analysis of monkey EBR study data

EBR data were first converted to blinks per minute. EBR were averaged by binning into 3 periods: Period 1 (0–1 h after the first dose) EBR = mean of 10, 20, 30, 40, and 50 min recordings; Period 2 (2–3 h after the first dose) EBR = mean of 130, 140, 150, 160, and 170 min recordings; Period 3 (5–6 h after the first dose) EBR = mean of 310, 320, 330, 340, and 350 min recordings. For Fig. 5c EBR data were averaged by binning data from periods 1 through 3 as daily EBR. Data from each animal was then normalized to their respective Day 1 EBR and expressed as percent of Day 1 EBR for each drug. EBR data were analyzed with SAS® 9.4 (SAS Institute Inc. Cary, NC) using a mixed linear model (Mixed Procedure) for repeated measures. The model included treatment, day, time period, and their interactions as fixed effects, and animal and animal-by-day interaction as random effects. A first order autoregressive model was used for longitudinal covariance structure. Differences between least squares means across days, overall and for each time period, were tested and adjusted for multiple comparisons via Tukey method. All tests were performed two-tailed at a 5% level of significance.

### Rat unilateral 6-OHDA lesion model of Parkinson's symptoms

Sprague-Dawley rats with 6-OHDA lesions were obtained from Taconic (Rensselaer, NY). For the lesion surgery rats (~175 g) were anesthetized with isoflurane and given desipramine to protect noradrenergic neurons (10 mg/kg). The medial forebrain was targeted using AP:−4.3 mm; ML:−1.2 mm; DV:8.3 mm using a 30 G cannula. A total of 14.3 u μg of 6-OHDA + 2% ascorbic acid in a volume of 4 μl was infused at 0.5 μl/min. The cannula was left in place post-infusion for 5 min. To assess the extent of the lesion animals were given an apomorphine (0.05 mg/kg S.C.) challenge 21 day after the lesion surgery. Matched sham lesioned animals from the same source received exactly the same procedures except saline was infused into the brain. Only animals with more than 180 contralateral rotations over 30 min or more than 30 rotations in multiple 5 min intervals were used for the study. Experiments began 12 weeks after lesion. Animals were adults (~20 weeks old) at the time of the rotation study with lesioned animals between 460–550 g and sham animals between 475–670 g.

### Rat 6-OHDA study testing procedure

Rats with sham (*n* = 20) and unilateral 6-OHDA lesions (*n* = 17) were obtained and four weeks after lesion used for the study. Animals were individually monitored and dosed in their Innovive (San Diego, CA) home cages (Outside dimensions: 17”L × 13.4”W × 7.8”H) that were in turn placed in custom-made isolation booths. Animals followed a normal light cycle (6:00 AM lights on and 6:00 PM lights off). Animals were provided with food and water ad libitum in the chambers. Activity was monitored with infrared ceiling cameras (Y-cam, London, UK) and rotational behavior was quantified by automated movie analysis via validated custom software. Animals were acclimated and recorded for 2 h to the test chambers prior to the first dose. During the first treatment period one group of animals received six doses of a catechol (A-77636, 0.32 mg/kg subcutaneously) and the other group received a non-catechol (PF-2334, 10.78 mg/kg, orally) administered every 12 h followed by one subcutaneous dose of the D2 agonist quinpirole (0.1 mg/kg s.c.). Following a two-week washout period, animals then received the other treatment (i.e., those who received A-77636 during the first treatment period received in the second treatment period six doses of PF-2234 administered every 12 h followed as well by a single dose of the the D2 agonist quinpirole and vice versa).

Pharmacokinetic modeling and simulations for rat 6-OHDA study dose selection and estimation of central receptor occupancy: Pharmacokinetic and neuropharmacokinetic studies were conducted in rats with A-77636 and PF-2334 to determine the plasma and brain concentration-time profiles (Fig. 1g, Supplementary Table 4, and additional data not shown). The resulting pharmacokinetic parameters were used to simulate doses for the rat 6-OHDA study. The objective of the modeling was to select the appropriate dose(s) and dose regimen to ensure that projected central receptor occupancy remained at or above a threshold value throughout the experiment. Expressing projected central receptor occupancy takes into account unbound brain concentrations (*C*_b,u_) and rat D1 binding Ki (see Supplementary Table 1). The projected central receptor occupancy was estimated through the use of *C*_b,u_ and the rat binding Ki at the D1 receptor through use of Eq. 2. The projected central receptor occupancy profiles of PF-2334 from the 10.8 mg/kg oral dose administered in the 6-OHDA study are illustrated in Supplementary Fig. 6. To avoid interfering with the animals behavior, no plasma concentration data was collected during the 6-OHDA study.

### 6-OHDA lesion study analysis

Rotational behavior was detected using custom software written in Python language, version 2.7. OpenCV library, version 2.10 was used for video analyses^73,74^. R software, version 3.1.0, was used for statistical analyses^70^. Rotational behavior is described as the average number of rotations per minute (RPM) during the 6 h period immediately following each dose. To quantify changes within each treatment group as well as differences between treatments over time we used a longitudinal mixed model two-way ANOVA^75^ with time, treatment, and their interaction as fixed factors and animal as a random factor. Least-squares means for specified factors were estimated using lsmeans library^76^. Post-hoc comparisons to reference time point were adjusted for multiple hypothesis testing via Dunnett′s method. All statistical tests were performed two-tailed at a 5% level of significance. Sham lesioned animals did not show any contralateral rotations with either of the D1R or D2R agonist doses (*n* = 20, data not shown). A-77636 treated 6-OHDA lesioned animals (*n* = 9) appeared to rotate more in response to the first agonist dose than the PF-2334 group (*n* = 8); however, this difference was not significant. For doses 2–4, the rotational behavior of both treatment groups overlapped extensively (Supplementary Fig. 5a). The rotational behavior of the two treatment groups began to diverge after the fourth dose (Fig. 5d). Post-hoc comparisons yielded both significant differences in the slopes (*t*(16) = 2.4, *p* < 0.029) as well as a significant difference in the number of rotations for animals treated with A-77636 vs. PF-2334 at 60 h (*t*(18) = 2.11, *p* < 0.049), indicating that the rotational behavior of PF-2334 treated animals stabilized whereas the rotational behavior of A-77636 treated animals continued to decrease (Supplementary Fig. 5). For A-77636 treated animals, D2R agonist treatment after the last D1R dose induced significantly greater rotational behavior (*t*(7) = 2.97, *p* < 0.021), indicating that the near absence of rotational behavior after the sixth A-77636 dose was not attributed to physical fatigue. Using a mixed model one-way ANOVA with time as fixed factor and animal as random factor we tested whether intensity of rotational motion is changing over time after repeated dosing with PF-2334. When all six PF-2334 doses are included along with D2 agonist treatment, time is significant *F*(6, 42) = 9.3, *p* < 1.7E-6. When the first PF-2334 dose and the D2 agonist treatment are excluded, time is no longer significant (*F*(3, 21) = 1.7, *p* < 0.2). At the same time, the model which excludes only the first two PF-2334 doses, yields significant effect of time (*F*(4,28) = 5.9, *p* < 0.0014). For this last model, pairwise post-hoc comparisons across time points computed via least-squares means and adjusted for multiple hypothesis testing via FDR correction reveal that only differences with D2 agonist treatment are significant, while pairwise differences across time points after PF-2334 doses at 24, 36, 48, 60 h are not (Supplementary Table 9). These data indicate that there is a more pronounced behavioral effect of the first dose of the non-catechol D1R agonist PF-2334 compared to subsequent doses, but from the second through the sixth dose, the behavioral response is stable and consistent with limited receptor desensitization.

### Synthetic chemistry and NMR validation of structures

Full methods are described in Supplementary Information.

### Data availability

The data that support the studies in this work are available from the corresponding author upon reasonable request.

## Electronic supplementary material

Supplementary Information

Description of Additional Supplementary Files

Supplementary Movie 1

Supplementary Movie 2

Supplementary Movie 3

Supplementary Movie 4

Supplementary Movie 5

Supplementary Movie 6

Supplementary Movie 7

Supplementary Movie 8

Supplementary Movie 9

Supplementary Movie 10

Supplementary Movie 11
