# Supplementary material for: Impaired β-arrestin recruitment and reduced desensitization by non-catechol agonists of the D1 dopamine receptor
Source: Nat Commun. 2018 Feb 14;9:674. doi: 10.1038/s41467-017-02776-7 (PMC5813016; doi:10.1038/s41467-017-02776-7)
Supplement: Supplementary file 1 — Supplementary Information [file 41467_2017_2776_MOESM1_ESM.pdf]

Supplementary Information

Supplementary Figures:

Supplementary Figure 1

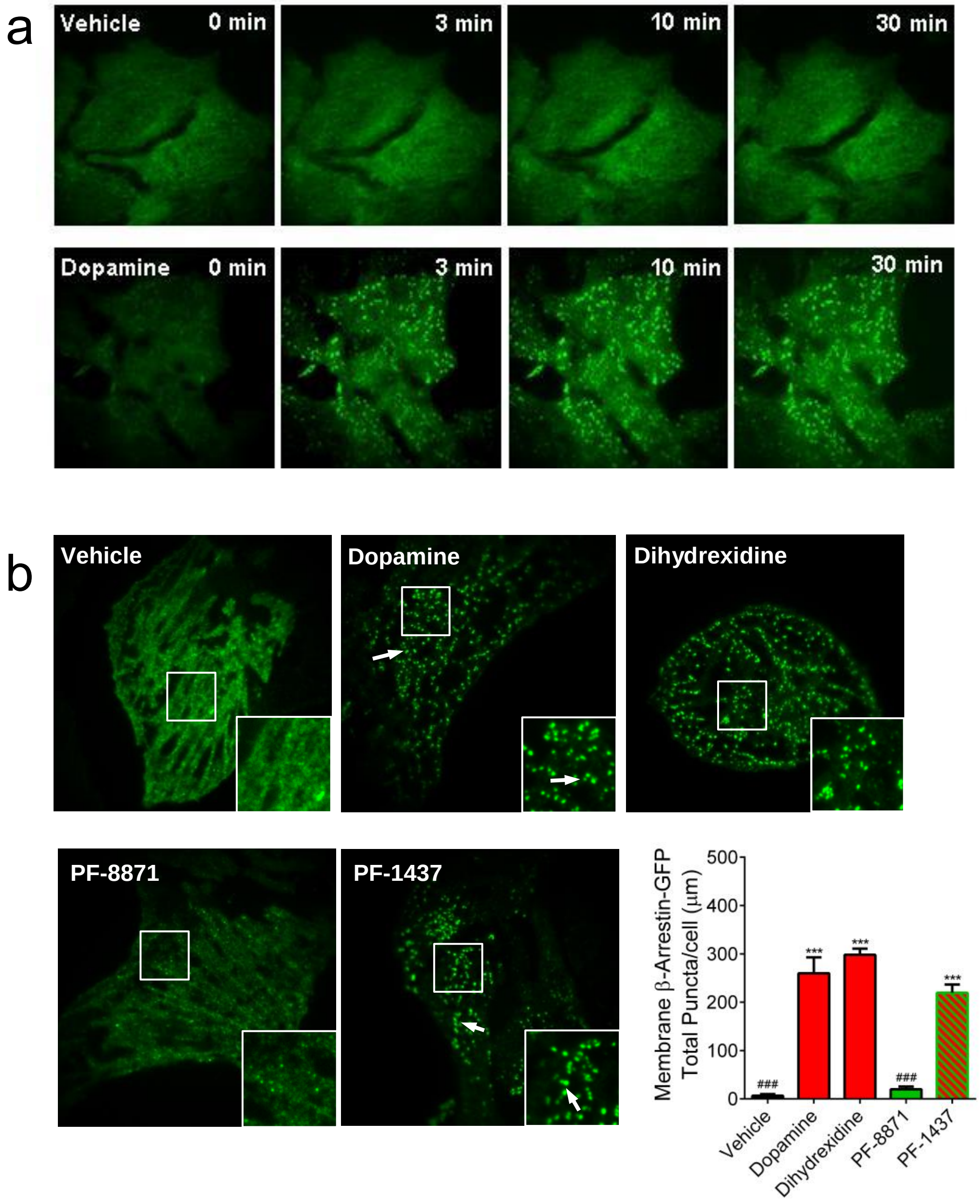

Supplementary Figure 1. DA-mediated β-arrestin membrane recruitment and conversion of non-catechol agonist PF-8871 into the catechol agonist PF-1437 reinstates β-arrestin membrane recruitment. a, Live-cell TIRFM images of β-arrestin-GFP at the plasma membrane

in U2OS cells at different time points following treatment with vehicle or dopamine (1 μM). **b**, TIRFM images and quantification of β-arrestin-GFP at the plasma membrane in U2OS cells after 10 min of treatment with vehicle or indicated agonists (1 μM), from ≥ 60 cells per group obtained across three independent experiments. Significant effect of treatment via one-way ANOVA ($F(5,15)=347$, $P<1.e-4$) with *post hoc* test relative to both vehicle and dopamine groups adjusted for multiple testing via FDR; significant contrasts with vehicle (*** $P<1.e-4$ for dopamine, dihydrexidine, and PF-1437) or dopamine (###, $P<1e-4$, for vehicle and PF-8871). Scale bars, 10 μm. All data are presented as means ± s.e.m.

Supplementary Figure 2

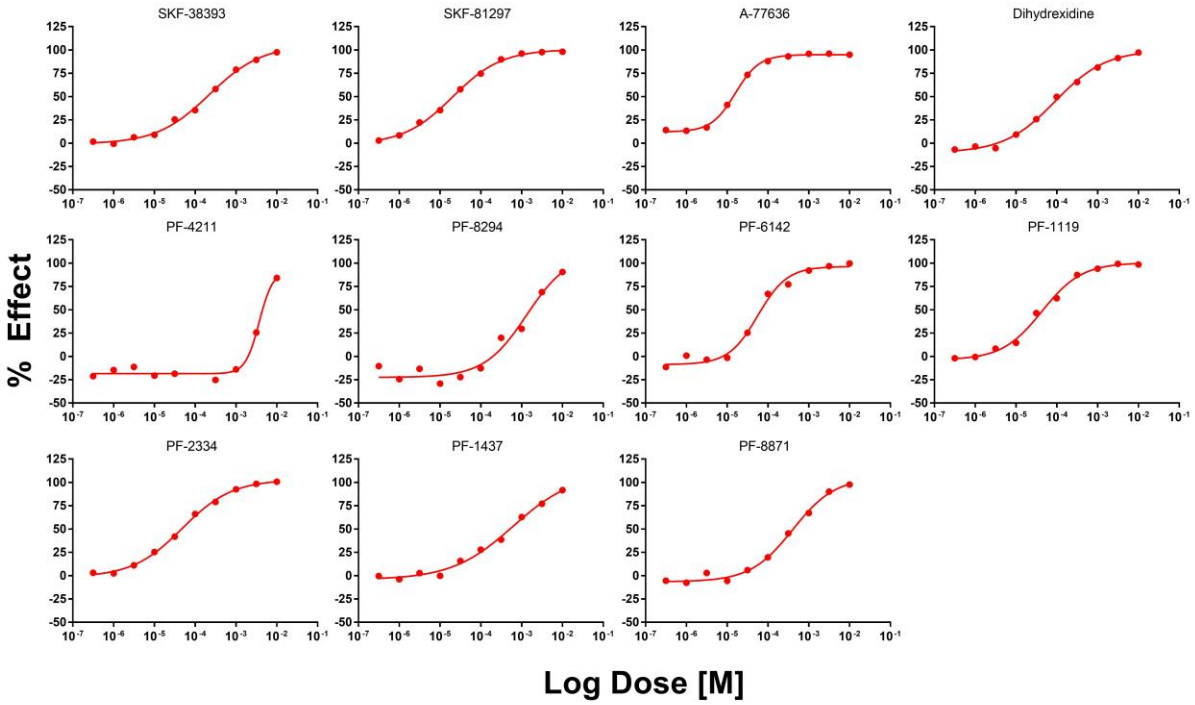

Supplementary Figure 2. A representative example of human D1 radioligand binding assay data and fitted curve for each of the 11 synthetic compounds in Supplementary Table 1.[a]

[a] human D1R radioligand binding assay conditions described in methods

Supplementary Figure 3

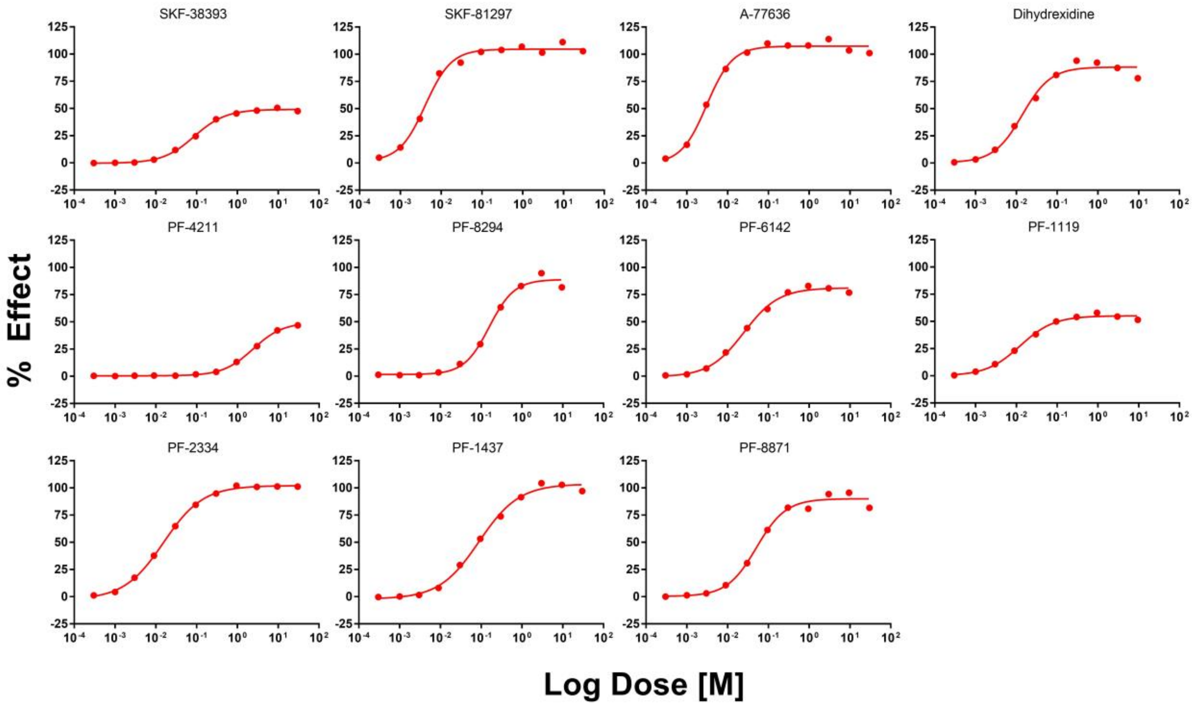

Supplementary Figure 3. A representative exemplar of human D1 cAMP assay experimental data and fitted curve for each of the 11 synthetic compounds in Supplementary Table 1.[a]

[a] human D1R functional cAMP assay conditions described in methods

Supplementary Figure 4

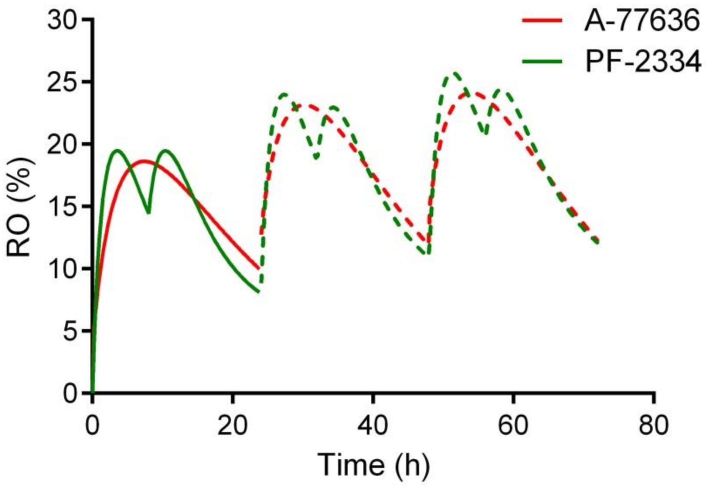

Supplementary Figure 4. Estimated D1 receptor occupancy by PF-2334 and A-77636 in monkey brain calculated using in-life plasma exposures from monkey eye blink rate experiment.

Supplementary Figure 5

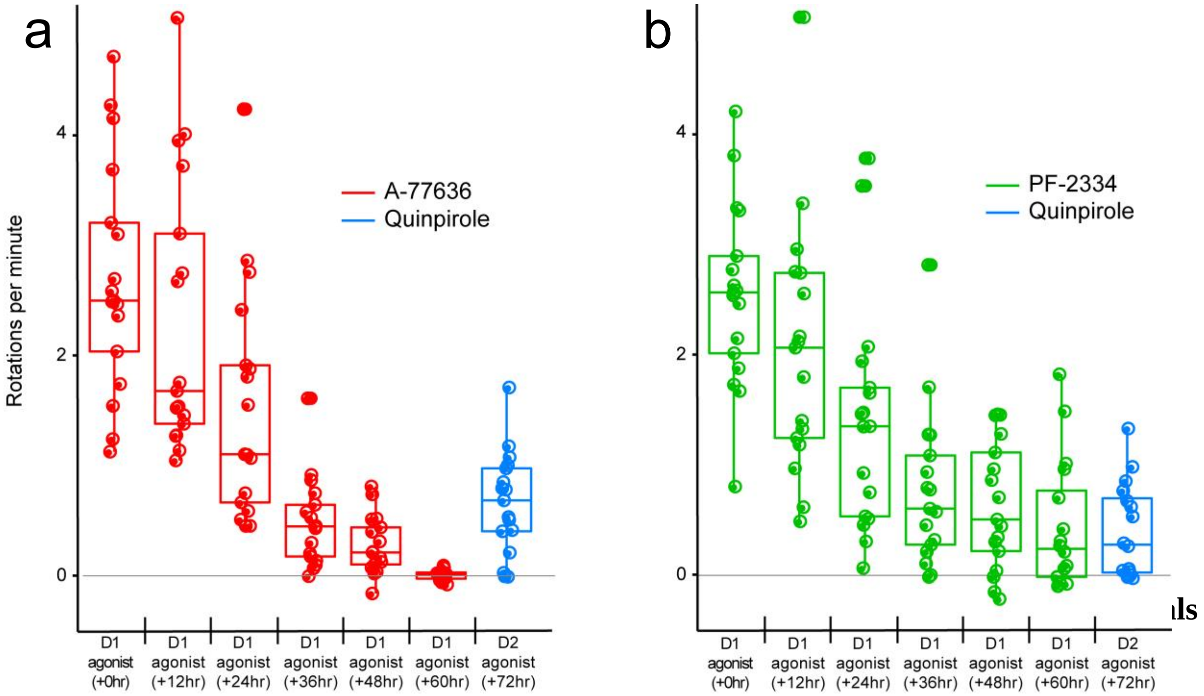

a, Red box plots represent average number of contralateral rotations/minute for each dose of A-77636 (0.32 mg/kg, s.c.) in 12 h bins with individual animal data shown for each period. Contralateral rotations/minute from a single dose of the D2 agonist quinpirole (0.1 mg/kg, s.c.) administered 72 hours after first D1 agonist dose is in blue. **b,** Green box plots represent average number of contralateral rotations/minute for each dose of PF-2334 (10.78 mg/kg, p.o.) in 12 h bins. Contralateral rotations/minute from a single dose of the D2 agonist quinpirole (0.1 mg/kg, s.c.) administered 72 hours after first D1 agonist dose is in blue.

Supplementary Figure 6

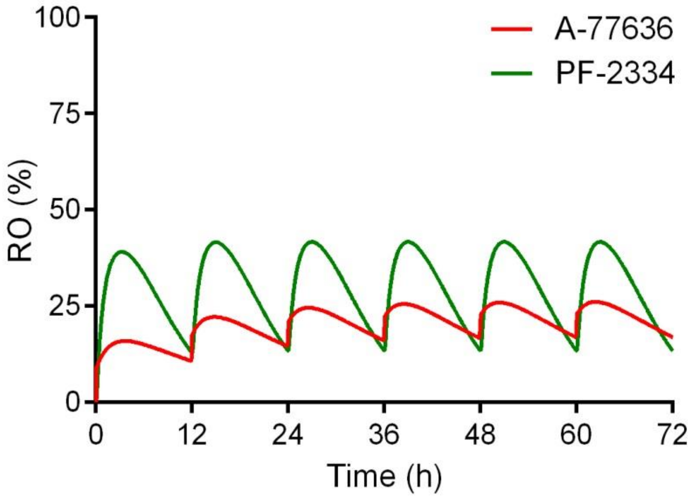

Supplementary Figure 6: Estimated D1 receptor occupancy in rat brain after repeated administration of A-77636 (0.32 mg/kg subcutaneously once/daily) or PF-2334 (10.8 mg/kg orally twice daily) calculated using parameters from rat neuropharmacokinetic assessment.

Supplementary Figure 7

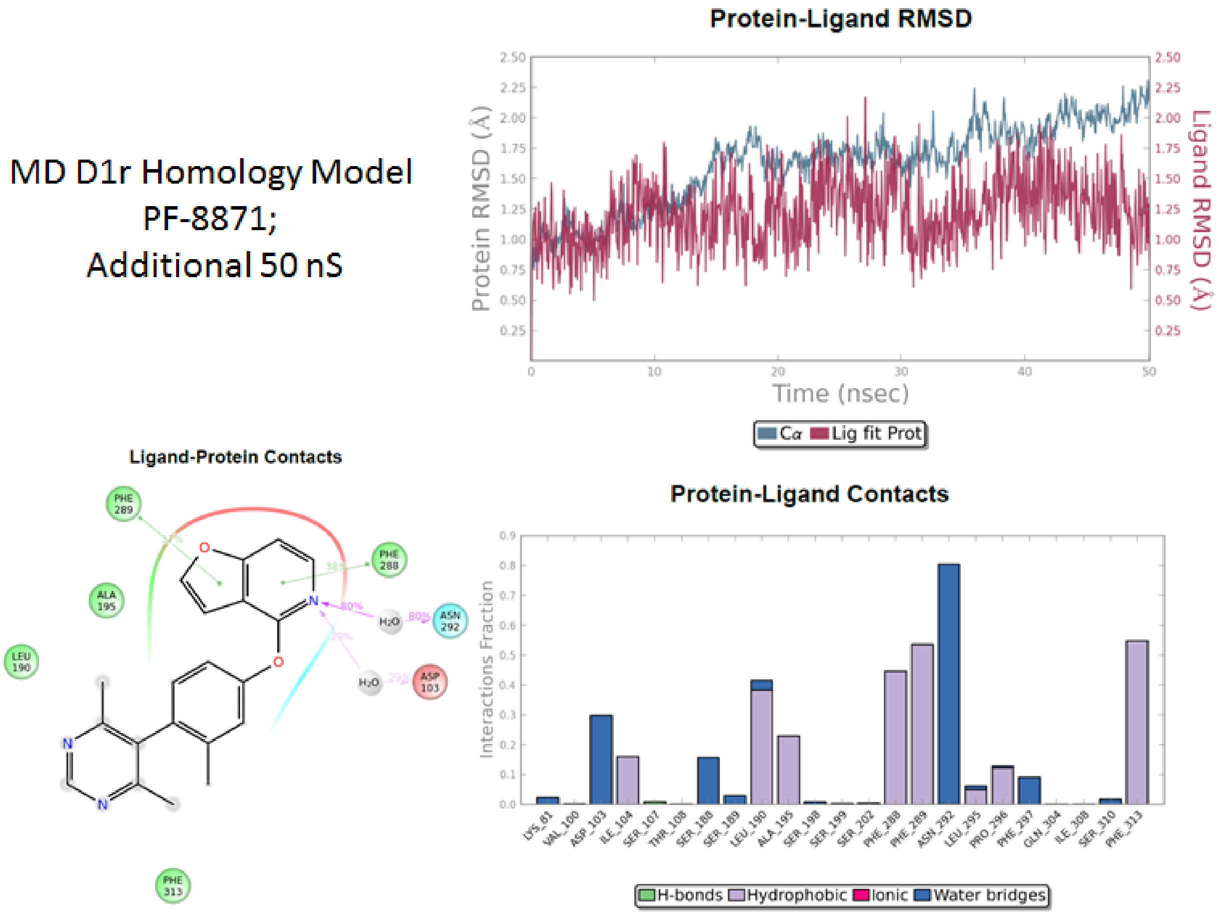

Supplementary Figure 7. Simulation Interaction diagram analysis of molecular dynamics simulations of PF-8871.

Supplementary Figure 8

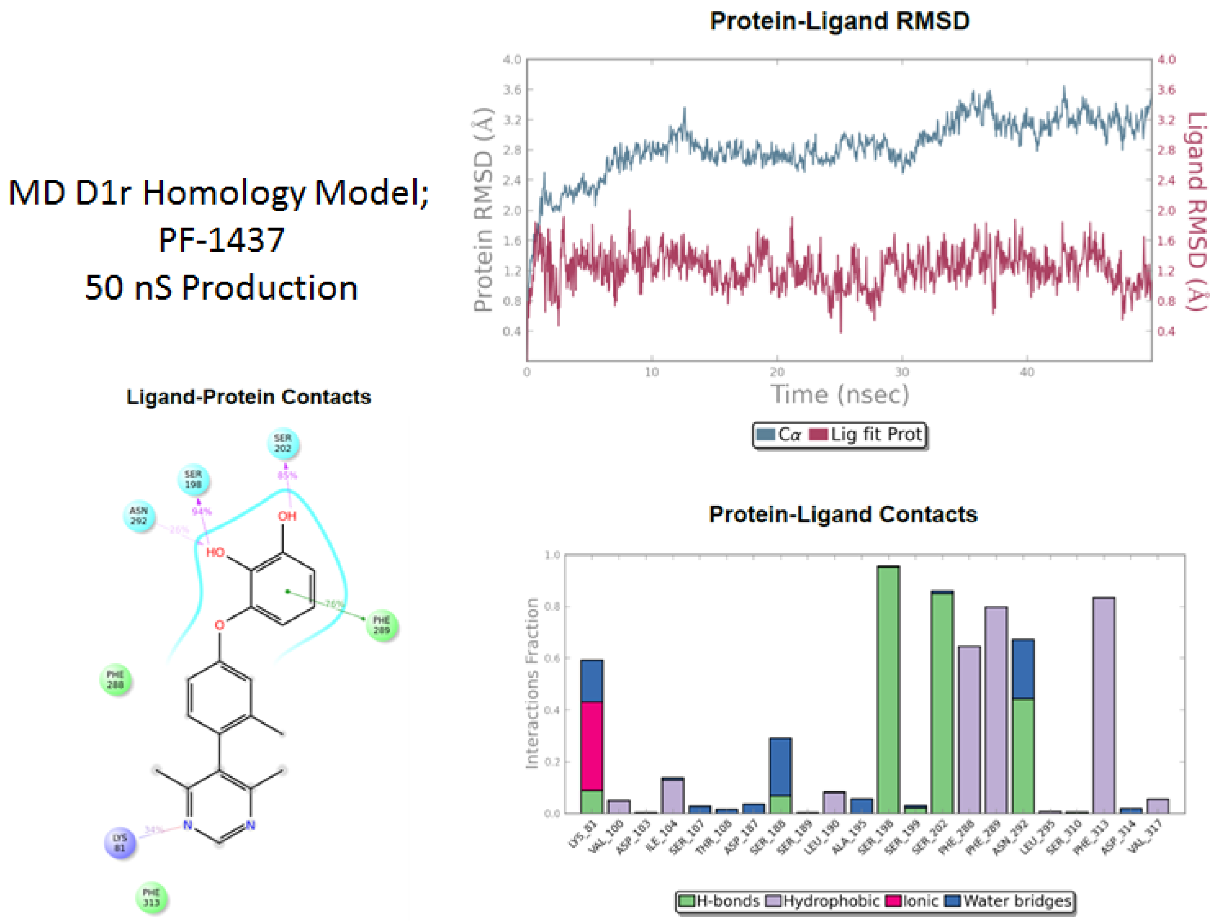

Supplementary Figure 8. Simulation Interaction diagram analysis of molecular dynamics simulations of PF-1437.

Supplementary Figure 9

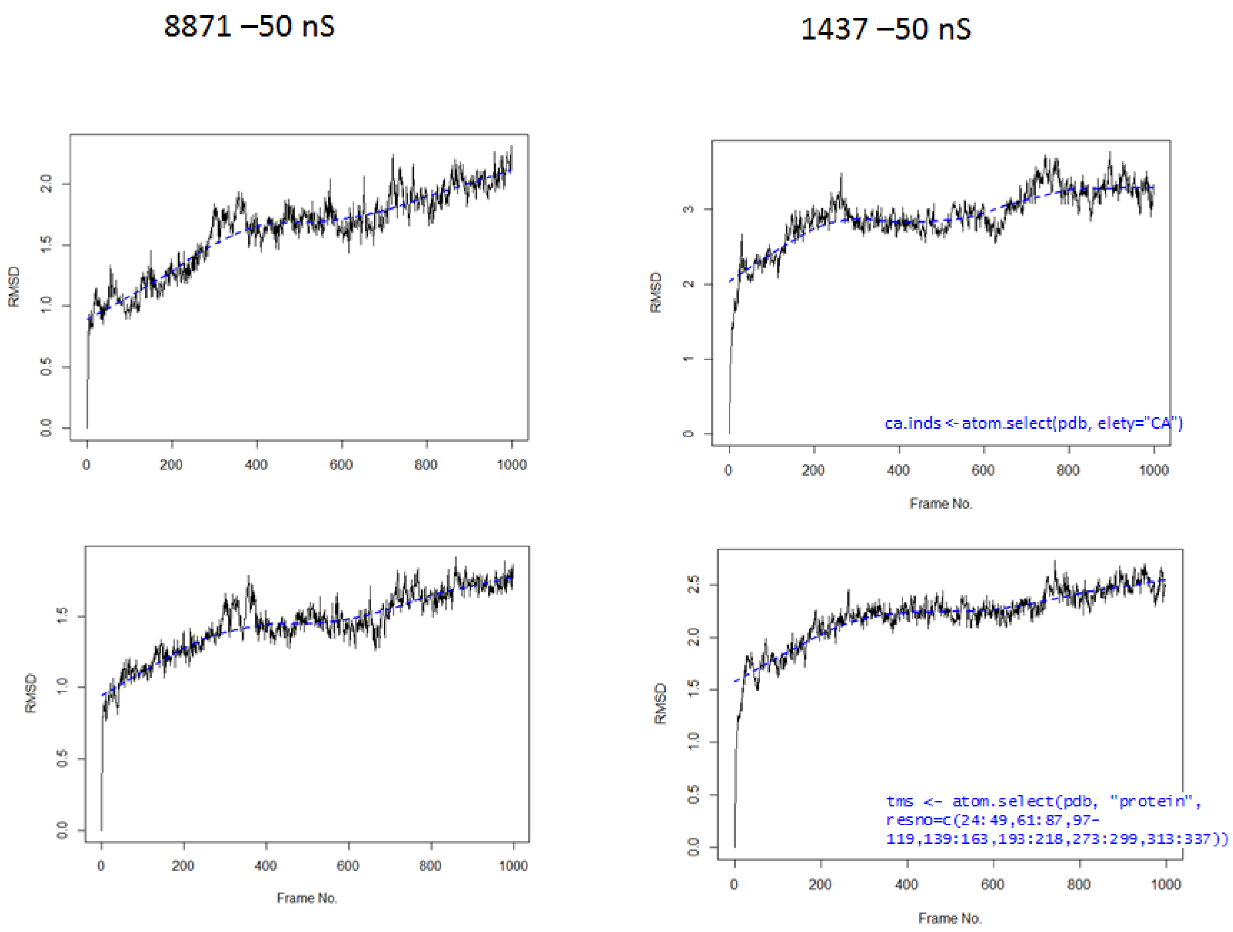

Supplementary Figure 9. RMSD plots for (top) all calpha atoms and (bottom) all protein transmembrane atoms.

Supplementary Figure 10

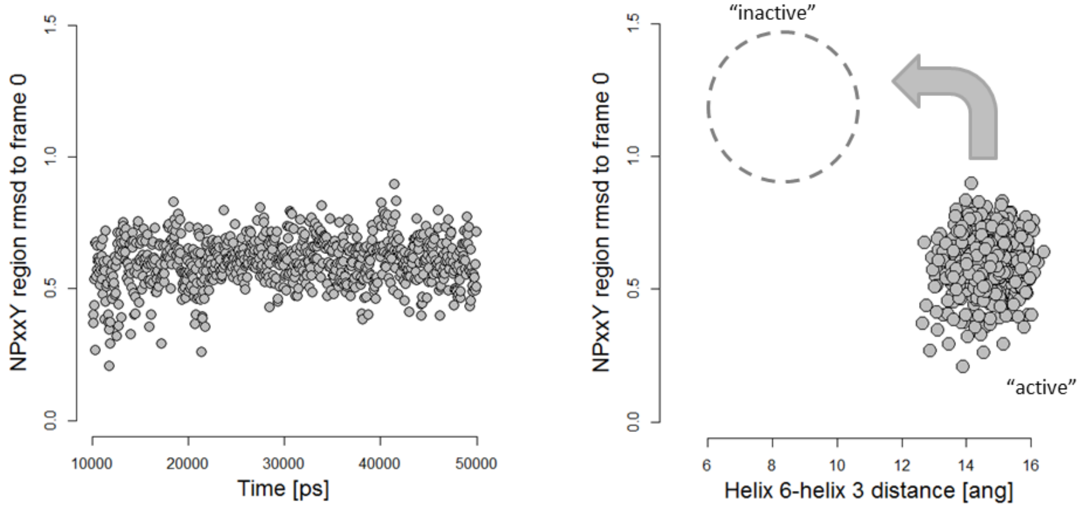

Supplementary Figure 10. Deactivation metrics[66] for the simulation of PF-8871. Left: the NPxxY (Asn322-Tyr326 in D1r) region rmsd to frame 0 (start of the averaging); and (right) helix 6-helix 3 distance as measured from the Ca atoms of Arg121 (helix 3) to Leu 271 (helix 6), vs. the NPxxY RMSD.

Supplementary Tables:

Supplementary Table 1: Dopamine receptor affinity and activity of catechol and non-catechol agonist ligands (mean from $n \geq 3$ experiments)[a, b]

	Human D1R Ki (nM)	Rat D1R Ki (nM)	Monkey D1R Ki (nM)	Potency hD1R cAMP EC50 (nM)	Efficacy hD1R cAMP	Human D5R Ki (nM)	Potency hD5R cAMP EC50 (nM)	Efficacy hD5R cAMP	Human D2R Ki (nM)	Human D3R Ki (nM)	Human D4R Ki (nM)
**Dopamine**	463±131	214±163	918±813	115±19	98±2	83±16	57±7	101±2	2539±673	160±8	20
**SKF-38393**	129±43	139±76	105±7	87±6	41±3	56±11	53±8	52±2	>4276	>671	nt
**SKF-81297**	22±2	9±2	6±1	4±1	98±1	11±3	2±0	100±1	>4276	>671	nt
**A-77636**	8±1	9±3	1±0.5	3±0.2	110±2	2±0.3	2±0	114±1	>4276	>671	nt
**Dihydrexidine**	64±6	nt	24±15	7±0.4	100±2	0.005±0.001	5±0.2	109±1	3718±300	387±21	nt
**PF-4211**	3136±335	nt	2565±416	2519±187	42±2	1891±508	1783±144	53±1	>4276	>671	nt
**PF-8294**	930±501	164±164	1436±703	153±17	78±4	276±220	95±8	83±1	>4276	>671	nt
**PF-6142**	40±6	18±4	66±9	17±2	92±3	14±2	9±1	89±1	>4276	>671	nt
**PF-1119**	26±3	nt	113±6	12±1	52±2	9±2	6±1	59±2	3398±366	>671	> 10000
**PF-2334**	51±13	nt	69±17	20±3	104±3	41±5	11±1	99±1	>4276	370±19	> 10000
**PF-1437**	729±150	nt	240±64	108±7	105±2	126±6	71±4	105±1	>4276	>701	> 10000
**PF-8871**	263±35	21±3	286±72	67±8	87±2	63±7	43±6	90±2	>4276	>671	>10000

[a]All data in the table generated as part of this work. Experimental conditions and data analysis are described in this supplement ,see sections on radioligand binding assays and cAMP assays.

[b]Supplementary Figures 2 and 3 depict a representative curve for Human D1R and HD1R cAMP assays respectively.

Supplementary Table 2. Selectivity Panel Results for non-catechol D1 agonists.

	Target	PF-6142	PF-8294	PF-8871	PF-1119
	Serotonin 2b, Agonist EC50 (nM)	>10,000	>10,000	>10,000	>10,000
	Muscarinic 1, Agonist EC50 (nM)	>10,000	>10,000	>10,000	>10,000
	Mu Opioid, Agonist EC50 (nM)	>10,000	>10,000	>10,000	>10,000
	Histamine 1, Agonist EC50 (nM)	>10,000	>10,000	>10,000	>10,000
	Cannabinoid 1, Agonist EC50 (nM)	>10,000	>10,000	>10,000	>10,000
	Adrenergic Beta 2, Agonist EC50 (nM)	>10,000	>10,000	>10,000	>10,000
	Adrenergic Alpha 1a, Agonist EC50 (nM)	>10,000	>10,000	>10,000	>10,000
GPCR Antagonist Selectivity Data	Dopamine 1, Antagonist IC50 (nM)	>10,000	>10,000	>10,000	>10,000
	Serotonin 2b, Antagonist IC50 (nM)	>10,000	>10,000	>10,000	>10,000
	Muscarinic 1, Antagonist IC50 (nM)	4,899	>10,000	>10,000	>10,000
	Mu Opioid, Antagonist IC50 (nM)	8,683	>10,000	>10,000	>10,000
	Histamine 1, Antagonist IC50 (nM)	4,557	>10,000	>10,000	>10,000
	Cannabinoid 1, Antagonist IC50 (nM)	2,080	>8762.7	>10000.0	6,604
	Adrenergic Beta 2, Antagonist IC50 (nM)	>10,000	>10,000	>10,000	>10,000
	Adrenergic Alpha 1a, Antagonist IC50 (nM)	9,103	>10,000	>10,000	>10,000
Ion Channel Data	Nav1.5 Sodium Channel Antagonist IC50 (nM)	1,094	>5895	8,679	2,643
	Nav1.5 Sodium Channel Agonist EC50 (nM)	>10,000	>10,000	>10,000	>10,000
	L-Type Calcium Channel, Agonist EC50 (nM)	>20,000	>20,000	>20,000	>20,000
	L-Type Calcium Channel Antagonist IC50 (nM)	3,253	>8,492	8,818	>10,000
Transporter Data	Dopamine Transporter, Inhibitor IC50 (nM)	>10,000	>10,000	>10,000	>10,000
	Norepinephrine Transporter, Inhibitor IC50 (nM)	>10,000	>10,000	>10,000	>10,000
	Serotonin Transporter, Inhibitor IC50 (nM)	>10,000	>10,000	>10,000	>10,000
PDE Data	Human PDE5A1 IC50 (nM)	>10,000	>10,000	>10,000	>10,000
	Human PDE4D3 IC50 (nM)	>10,000	>10,000	>10,000	1,806
	Human PDE3A1 IC50 (nM)	5,119	>10,000	>10,000	9,205

Supplementary Table 3: Ligand selected pharmacokinetic parameters measured in rat

Compound	PK Parameter				
	CL (mL/min/kg)	Vdss (L/kg)	t½ (h)	Cmax (ng/mL)	F (%)
SKF-38393	245	3.9	0.4	49.8	9.5
PF-6142	42.5	2.4	1.1	326	85.4
PF-2334	20.8	1.2	0.8	202	57.2

Supplementary Table 4: Average computed angle and distance between ligand and residue (Å)

Domain	Residue	Angle (degrees)	Dopamine (Å)	PF-6142 (Å)	PF-8871 (Å)	PF-1437 (Å)
**TM3**	**D103**	0	2.6	3.6	3.9	4.3
**ECL2**	**S188**	60	11.1	3	2.4	2.3
**ECL2**	**L190**	110	7.2	3.5	3.5	3.6
**TM5**	**S198**	180	2.9	4.8	4.7	2.9
**TM5**	**S202**	210	2.9	4.2	4.5	2.8

Supplementary Table 5: Saturation Binding Data Analysis

Compound		Bmax Estimate	Bmax std dev.	Bmax Diff. to Total	Bmax Diff. Std. Dev	Bmax Lower 95% Conf. Int.	Bmax Upper 95% Conf. Int.	Kd Estimate	Kd std dev	Kd Diff. to Total	Kd Diff. Std. Dev	Kd Lower 95% Conf. Int.	Kd Upper 95% Conf. Int.
PF-6142	Total	4211	78	-	-	-	-	0.94	0.13	-	-	-	-
PF-6142	3.2nM	4074	81	136	92	-42	320	1.93	0.17	-0.99	0.12	-1.23	-0.76
PF-6142	10.0nM	3771	82	439	94	246	621	1.82	0.17	-0.88	0.13	-1.14	-0.64
PF-8871	Total	4376	78	-	-	-	-	1.14	0.13	-	-	-	-
PF-8871	32nM	4001	80	375	88	199	546	2.61	0.18	-1.47	0.14	-1.77	-1.20
PF-8871	100nM	3728	80	648	90	468	820	2.81	0.20	-1.67	0.17	-2.01	-1.36
PF-1437	Total	4393	80	-	-	-	-	1.28	0.13	-	-	-	-
PF-1437	100nM	4069	82	324	90	143	501	2.89	0.20	-1.60	0.16	-1.90	-1.30
PF-1437	320nM	3635	84	758	100	558	951	3.06	0.23	-1.78	0.20	-2.19	-1.42

Supplementary Table 6: D1R agonist fold shift of cAMP functional potency in D1R single-residue mutants relative to wild-type (EC_{50} mutant/ EC_{50} WT).

Test Compound	Experiment #	T59A	K81R	D103A	S107A	I117A	S188A	S188I	S188T	L190A	Y194F	S198A	S199A	S202A	V270A	T273A	F288A	F289A	N292A	W318A	W321A	N334A
Dopamine	1	0.2	1.1	A.L.D.	15.3	25.6	0.3	1.6	1.0	123.2	1.4	15.9	28.7	52.7	1.0	0.3	441.8	304.0	A.L.D.	21.2	9.4	0.9
	2			A.L.D.			4.5	4.1	5.2	53.0	3.4	32.0	42.2	249.2			A.L.D.	282.5	A.L.D.	16.0	12.7	
	3			A.L.D.			1.4	2.7	4.7	54.2	1.6	20.0	28.4	36.4			91.6	30.5	A.L.D.	14.2	11.8	
	4			A.L.D.			0.3	1.1	0.6	12.4	1.2	19.1	5.6	11.3			A.L.D.	41.8	34.7	6.5	7.3	
	5			A.L.D.				3.3		36.4		140.5		76.3								
	6			A.L.D.				6.2		37.7		13.4		60.0								
SKF-38393	1	0.5	1.2	A.L.D.	5.8	7.9	0.5	0.2	0.4	906.0	0.4	8.4	10.3	3.6	0.8	0.4	48.7	18.7	181.6	9.1	8.9	1.5
	2			A.L.D.			0.9	0.4	0.4	358.1	1.3	A.L.D.	13.7	2.7			41.1	40.0		7.7	12.5	
	5			A.L.D.				0.2		214.0		23.9		6.1								
	6			A.L.D.				0.4		147.0		6.3		2.9								
SKF-81297	3			1495.0			0.1	0.1	0.0	16.7	17.3	11.3	1.5	0.1			81.0	8.6	217.3	1.1	5.3	
	5			3588.0				0.9		231.0		68.6		69.4								
	6			1854.8				1.2		174.0		20.1		40.6								
Dihydrexidine	1	0.2	1.1	147.1	5.2	14.2	0.4	0.7	0.4	204.0	0.9	23.1	21.0	21.0	2.9	0.7	735.7	16.4	34.0	15.4	4.7	1.2
	2			180.6			0.7	0.9	0.4	278.0	3.4	49.8	39.7	75.3			930.7	64.0	497.0	8.1	20.6	
	3			673.5			0.5	1.5	0.5	372.0	2.8	38.0	91.5	143.0			425.3	67.5	466.3	30.5	11.3	
	5			669.1				0.9		231.0		68.6		69.4								
	6			973.4				1.2		174.0		20.1		40.6								
A-77636	1	0.2	0.1	45.6	2.4	5.8	0.1	0.1	0.1	9.0	0.2	5.9	6.4	12.3	0.6	0.2	735.7	16.4	34.0	15.4	4,67	1.2
	2			85.9			0.6	0.2	1.6	8.6	0.6	19.0	6.6	17.8			930.7	64.0	497.0	8.1	20.6	
	3			112.9			0.3	0.1	0.1	11.7	1.6	10.4	24.9	21.4			425.3	67.5	466.3	30.5	11.3	
PF-2334	1	B.L.D.	1.3	34.0	2.3	8.3	1.0	4.0	1.7	522.7	1.3	6.0	8.0	1.7	1.3	0.7	351.3	67.3	51.0	12.0	12.3	1.3
	4			31.0			0.4	6.0	2.0	467.0	1.0	8.0	4.0	1.0			522.0	77.0	27.0	18.0	19.0	
	5			35.9				6.7		1180.0		11.5		1.9								
	6			43.9				5.6		498.0		7.2		1.0								
PF-6142	1	1.0	2.7	31.3	2.3	9.8	2.0	6.7	1.7	153.2	1.7	6.0	8.7	1.2	1.7	0.2	141.2	32.3	23.7	16.3	22.7	1.3
	3			42.6			2.3	10.6	1.3	155.7	0.8	7.0	10.0	1.6			88.7	44.7	30.6	20.4	21.1	
	5			78.1				8.5		225.0		6.4		2.8								
	6			60.6				10.1		696.0		7.5		1.9								
PF-8871	4			12.6			0.6	8.7	1.8	134.4	1.3	3.3	4.3	1.7			146.3	27.9	30.7	12.5	21.4	
	5			34.4				5.8		522.0		5.7		2.6								
	6			39.7				10.8		598.0		8.6		1.7								
PF-1437	1	0.2	0.4	0.5	1.2	15.2	0.7	1.4	0.9	63.9	0.3	8.1	7.9	15.3	0.6	0.1	61.0	12.4	19.1	8.3	1.8	1.2
	2			0.4			1.0	1.8	0.6	20.0	0.4	A.L.D.	10.5	15.9			50.5	16.0	A.L.D.	3.5	1.2	
	3			0.4			0.8	4.7	0.9	62.5	1.9	41.6	34.1	30.1			139.0	16.0	93.7	7.8	5.4	
	5			0.7				4.8		46.7		20.5		23.1								

A.L.D. = above limit of detection
B.L.D. = below limit of detection

Supplementary Table 7: Mean (± standard deviation) plasma concentrations of PF-2334 in monkeys undergoing an eye blink rate (EBR) study following 0.9 mg/kg/day split oral dose (0.6 mg/kg followed 8 h later by 0.3 mg/kg)

Day	Time post-dose (h)	C_p (ng/mL)	$C_{p,u}$ (nM)
1	1	41.9 ± 9.4	4.8 ± 1.1
1	5	47.0 ± 13.9	5.4 ± 1.6
2	1	71.4 ± 20.1	8.1 ± 2.3
2	5	99.0 ± 36.3	11.3 ± 4.1
3	1	89.5 ± 49.4	10.2 ±5.6
3	5	106 ± 40	12.1 ± 4.5

C_p (total plasma concentration)
$C_{p,u}$ (unbound plasma concentration)

Supplementary Table 8: Mean (± standard deviation) plasma concentrations of A-77636 in monkeys undergoing an EBR study after 1 mg/kg/day subcutaneous dose

Day	Time post-dose (h)	C_p (ng/mL)	$C_{p,u}$ (nM)
1	1	79.3 ± 11.4	10.6 ± 1.5
1	6	34.8 ± 3.5	4.6 ± 0.5
2	1	73.1 ± 13.8	9.8 ± 1.8
2	6	46.3 ± 11.7	6.2 ± 1.6
3	1	85.5 ± 19.0	11.4 ± 2.5
3	6	40.6 ± 2.8	5.4 ± 0.4

C_p (total plasma concentration)
$C_{p,u}$ (unbound plasma concentration)

Supplementary Table 9: 6-OHDA data analysis**.** Post-hoc pairwise comparisons of rotational speed for PF-2334 doses 2-6 and quinpirole dose across different time intervals

contrast (time range, hours)	estimate	SE	df	t.ratio	p.value.fdr
24 - 36	0.4016259	0.23244	28	1.728	0.1901
24 - 48	0.33219881	0.23244	28	1.429	0.2343
24 - 60	0.37097004	0.23244	28	1.596	0.2029
24 - 72	1.09306565	0.23244	28	4.703	0.0006
36 - 48	-0.06942709	0.23244	28	-0.299	0.896
36 - 60	-0.03065587	0.23244	28	-0.132	0.896
36 - 72	0.69143975	0.23244	28	2.975	0.015
48 - 60	0.03877122	0.23244	28	0.167	0.896
48 - 72	0.76086684	0.23244	28	3.273	0.0141
60 - 72	0.72209561	0.23244	28	3.107	0.0144

computed via least-squares means and adjusted for multiple hypothesis testing via FDR correction

Supplementary Methods:

Synthetic chemistry and NMR validation of structures

PF-4211

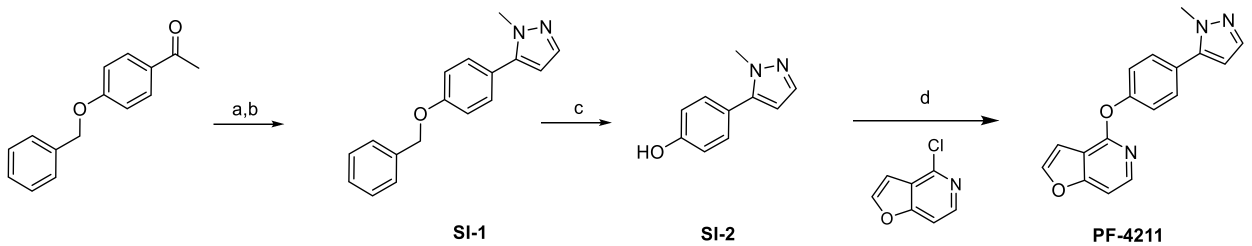

SI-1 SI-2 PF-4211

Reagents and conditions: (a) DMF dimethyl acetal, DMF, reflux; (b) methylhydrazine, 75 ^{0}C; (c) H_2, Pd/C; (d) NaH, DMF, 100 ^{0}C.

5-[4-(benzyloxy)phenyl]-1-methyl-1H-pyrazole (SI-1)

N,N-Dimethylformamide dimethyl acetal (94%, 19.0 mL, 134 mmol) was added to a solution of 1-[4-(benzyloxy)phenyl]ethanone (15.32 g, 67.71 mmol) in *N,N*-dimethylformamide (30 ml) and the reaction mixture was heated at reflux for 18 h. At this point, the reflux condenser was replaced with a distillation head, and distillation was carried out until the temperature of the distillate reached 140ºC. The material in the reaction pot was cooled to room temperature, treated with methylhydrazine (98%, 7.4 mL, 136 mmol) and heated at 75 ºC for 3 h. The reaction

mixture was cooled, diluted with ethyl acetate, washed four times with aqueous 5% NaCl solution, dried over $MgSO_4$, filtered, and concentrated *in vacuo*. Purification via silica gel chromatography (Gradient: 2% to 10% ethyl acetate in dichloromethane) yielded the product as a light yellow solid. Yield: 13.79 g, 52.17 mmol, 77%. LCMS *m/z* 265.1 (M+H). ^{1}H NMR (400 MHz, DMSO-d_6) characteristic peaks, δ 3.81 (s, 3H), 5.17 (s, 2H), 6.31 (d, *J*=1.5 Hz, 1H), 7.12 (d, *J*=8.8 Hz, 2H).

4-(1-methyl-1H-pyrazol-5-yl)phenol (SI-2)

5-[4-(Benzyloxy)phenyl]-1-methyl-1*H*-pyrazole (**SI-1**) (13.49 g, 51.04 mmol) was mixed with 10% palladium on carbon (~50% in water, 1.46 g) and dissolved in ethanol (125 ml). The reaction mixture was hydrogenated at room temperature and 1 atmosphere hydrogen for 18 h, then filtered and concentrated *in vacuo*. The residue was triturated with heptane to yield the product as a colorless solid. Yield: 8.74 g, 50.2 mmol, 98%. LCMS *m/z* 175.1 (M+H). ^{1}H NMR (400 MHz, DMSO-d_6) δ 9.73 (br s, 1H), 7.40 (d, *J*=1.9 Hz, 1H), 7.31 (br d, *J*=8.7 Hz, 2H), 6.86 (br d, *J*=8.7 Hz, 2H), 6.26 (d, *J*=1.9 Hz, 1H), 3.79 (s, 3H).

4-[4-(1-methyl-1H-pyrazol-5-yl)phenoxy]furo[3,2-c]pyridine (PF-4211).

4-(1-Methyl-1*H*-pyrazol-5-yl)phenol (**SI-2**) (25 mg, 0.14 mmol) was dissolved in 1 ml of DMF. Soduim hydride (6.9 mg, 0.17 mmol) was added and the reaction mixture was stirred for 1 h at room temperature. 4-Chlorofuro[3,2-*c*]pyridine (22 mg, 0.14 mmol) was added and the reaction mixture was stirred at 100 ^{0}C overnight. After cooling to room temperature, the reaction mixture was diluted with methanol and evaporated i*n vacuo*. The crude material was initially purified using silica gel chromatography (Eluent: 50% ethyl acetate in heptane with gradient to 100% ethyl acetate) to afford a mixture of the final product and the starting phenol. The desired material was isolated by preparataive TLC (silica gel; eluent: 5% methanol in dichloromethane). Yield: 9 mg (20%). LCMS *m/z* 291.9 (M+H). ^{1}H NMR (500 MHz, CD_3OD) δ 7.96 (d, *J*=6.1 Hz, 1 H), 7.89 (d, *J*=2.1 Hz, 1 H), 7.56 (d, *J*=8.6 Hz, 2 H), 7.51 (d, *J*=2.0 Hz, 1 H), 7.37 (dd, *J*=6.0, 0.9 Hz, 1 H), 7.32 (d, *J*=8.8 Hz, 2 H), 6.93 (dd, *J*=2.2, 0.9 Hz, 1 H), 6.39 (d, *J*=2.0 Hz, 1 H), 4.86 (s, 3 H), 3.91 (s, 3 H). ^{13}C NMR (101 MHz, $CDCl_3$) δ ppm 162.12, 157.34, 153.89, 144.75, 142.94, 141.70, 138.47, 130.00, 127.17, 121.47, 113.09, 106.07, 104.08, 103.84, 37.48.

PF-8871

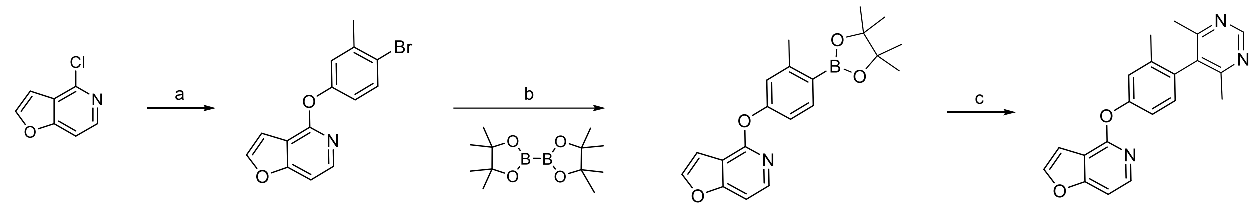

SI-3 SI-4

Reagents and conditions: (a) 4-bromo-3-methylphenol, DMSO, Cs_2CO_3, 135°C; (b) Pd(dppf)Cl_2, KOAc, dioxane; (c) 5-bromo-4,6-dimethylpyrimidine, $Pd_2(dba)_3$, PCy_3, K_3PO_4, dioxane/water.

4-(4-bromo-3-methylphenoxy)furo[3,2-c]pyridine (SI-3)

To a solution of 4-chlorofuro[3,2-c]pyridine (120 g, 781 mmol) in dimethyl sulfoxide (1.56 L), was added cesium carbonate (509 g, 1.56 mol) and 4-bromo-3-methylphenol (161 g, 861 mmol), and the reaction was heated to 125°C for 16 h. At this point, the reaction mixture was cooled to room temperature, poured into water (5 L), and extracted with ethyl acetate (2 x 2.5 L). The combined organic extracts were washed with water (2.5 L), washed with saturated aqueous sodium chloride solution (2.5 L), dried over anhydrous sodium sulfate, filtered and concentrated *in vacuo*. Purification by chromatography on silica gel (Eluent: 2% ethyl acetate in petroleum ether) yielded the product as a pale yellow solid. Yield: 205 g, 674 mmol, 86%. LCMS *m/z* 304.0, 306.0 (M+H). ^{1}H NMR (400 MHz, $CDCl_3$) δ 8.00 (d, *J*=6.2 Hz, 1H), 7.64 (d, *J*=2.1 Hz, 1H), 7.55 (d, *J*=8.3 Hz, 1H), 7.20 (dd, *J*=5.8, 0.8 Hz, 1H), 7.12 (d, *J*=2.9 Hz, 1H), 6.93 (dd, *J*=8.5, 2.7 Hz, 1H), 6.88 (dd, *J*=2.5, 0.8 Hz, 1H), 2.41 (s, 3H).

4-[3-methyl-4-(4,4,5,5-tetramethyl-1,3,2-dioxaborolan-2-yl)phenoxy]furo[3,2-c]pyridine (SI-4)

To a stirred solution of 4-(4-bromo-3-methylphenoxy)furo[3,2-*c*]pyridine (**SI-3**) (50.0 g, 164 mmol) in 1,4-dioxane (1.02 L) was added 4,4,4',4',5,5,5',5'-octamethyl-2,2'-bi-1,3,2-dioxaborolane (41.76 g, 164.4 mmol), potassium acetate (64.6 g, 658 mmol) and [1,1'-bis(diphenylphosphino)ferrocene]dichloropalladium(II) (6.0 g, 8.2 mmol), and the reaction mixture was heated at 85 °C for 16 h. After cooling to room temperature, the reaction mixture was filtered through a pad of Celite, and the pad was washed with ethyl acetate. The combined filtrates were concentrated *in vacuo* and the residue was purified by silica gel chromatography

(Eluent: 2% ethyl acetate in petroleum ether) to provide the product as a white solid. Yield: 40.0 g, 114 mmol, 70%. LCMS *m/z* 352.2 (M+H). ^{1}H NMR (400 MHz, $CDCl_3$) δ 8.02 (d, *J*=5.8 Hz, 1H), 7.84 (d, *J*=7.5 Hz, 1H), 7.61 (d, *J*=2.1 Hz, 1H), 7.19 (d, *J*=5.8 Hz, 1H), 7.00 (m, 2H), 6.80 (m, 1H), 2.56 (s, 3H), 1.34 (s, 12H).

4-[4-(4,6-dimethylpyrimidin-5-yl)-3-methylphenoxy]furo[3,2-c]pyridine (PF-8871)

4-[3-Methyl-4-(4,4,5,5-tetramethyl-1,3,2-dioxaborolan-2-yl)phenoxy]furo[3,2-*c*]pyridine (**C2**) (250 mg, 0.712 mmol), 5-bromo-4,6-dimethylpyrimidine (160 mg, 0.855 mmol), tris(dibenzylideneacetone)dipalladium(0) (95%, 26.9 mg, 0.142 mmol), tricyclohexylphosphine (79.9 mg, 0.285 mmol) and potassium phosphate (302 mg, 1.42 mmol) were combined in a 3:1 mixture of 1,4-dioxane and water (12 ml), and subjected to irradiation in a microwave reactor at 120ºC for 5 h. The reaction mixture was filtered through Celite, and the filtrate concentrated under reduced pressure, taken up in ethyl acetate, filtered through silica gel (1 g), and concentrated *in vacuo*. Purification via silica gel chromatography (Gradient: 0% to 100% ethyl acetate in heptane) yielded the product as a colorless oil. Yield: 123 mg, 0.371 mmol, 52%. LCMS *m/z* 332.1 (M+H). ^{1}H NMR (500 MHz, $CDCl_3$) δ 8.98 (s, 1H), 8.07 (d, *J*=5.9 Hz, 1H), 7.67 (d, *J*=2.2 Hz, 1H), 7.25-7.27 (m, 1H, assumed; partially obscured by solvent peak), 7.24 (br d, *J*=2.4 Hz, 1H), 7.19 (br dd, *J*=8.3, 2.4 Hz, 1H), 7.08 (d, *J*=8.3 Hz, 1H), 6.90 (dd, *J*=2.2, 1.0 Hz, 1H), 2.27 (s, 6H), 2.04 (s, 3H). ^{13}C NMR (101 MHz, $CDCl_3$) δ ppm 164.91, 162.18, 157.26, 156.85, 153.97, 144.72, 141.80, 137.31, 132.71, 131.94, 129.72, 122.72, 119.10, 113.28, 104.11, 103.87, 22.72, 19.67.

PF-8294

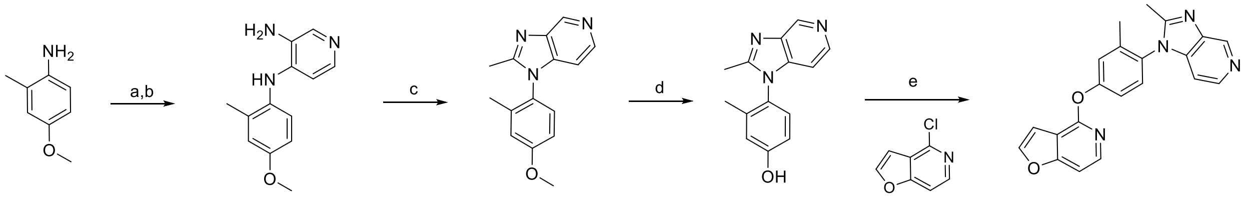

SI-5 SI-6 SI-7 PF-8294

Reagents and conditions: (a) 4-chloro-3-nitropyridine, triethylamine, EtOH, RT; (b) H_2, Pd/C, MeOH; (c) triethyl orthoacetate, Ac_2O; (d) BBr_3, dichloromethane; (e) DMSO, Cs_2CO_3, 140 ^{0}C.

N^4-(4-methoxy-2-methylphenyl)pyridine-3,4-diamine (SI-5)

A solution of 4-methoxy-2-methylaniline (23.8 g, 173 mmol), 4-chloro-3-nitropyridine (25 g, 160 mmol), and triethylamine (33.0 mL, 237 mmol) in ethanol (250 ml) was stirred at room temperature for 16 h, then concentrated under reduced pressure. The residue was dissolved in ethyl acetate (200 ml) and filtered through a thick pad of silica gel (Eluent: ethyl acetate, 1 L). The filtrate was concentrated *in vacuo* to provide N-(4-methoxy-2-methylphenyl)-3-nitropyridin-4-amine as a purple oil, which solidified on standing. This material was used without further purification. Yield: 41 g, 160 mmol, 100%. LCMS *m/z* 260.1 (M+H).

Palladium on carbon (10%, 3 x 2.12 g) was added to each of three batches of crude *N*-(4-methoxy-2-methylphenyl)-3-nitropyridin-4-amine (each approximately 10 g; total 31 g, 120 mmol) in methanol (3 x 100 mL). The three suspensions were independently hydrogenated under 45 psi hydrogen at room temperature on a Parr shaker for 24 h. The three reaction mixtures were combined, filtered through a pad of Celite, and concentrated *in vacuo*. Purification by silica gel chromatography [Gradient: 2% to 10% (1.7 M ammonia in methanol) in dichloromethane] yielded the product as a light brown solid. Yield: 24.0 g, 105 mmol, 88%. LCMS *m/z* 230.1 (M+H). ^{1}H NMR (400 MHz, $CDCl_3$) δ 8.01 (s, 1H), 7.88 (d, *J*=5.5 Hz, 1H), 7.08 (d, *J*=8.6 Hz, 1H), 6.84 (br d, *J*=2.8 Hz, 1H), 6.78 (br dd, *J*=8.6, 3.0 Hz, 1H), 6.34 (d, *J*=5.5 Hz, 1H), 5.66 (br s, 1H), 3.82 (s, 3H), 2.20 (br s, 3H).

1-(4-methoxy-2-methylphenyl)-2-methyl-1H-imidazo[4,5-c]pyridine (SI-6)

A mixture of *N*-(4-methoxy-2-methylphenyl)pyridine-3,4-diamine (**C7**) (3.95 g, 17.2 mmol), acetic anhydride (1.96 mL, 20.7 mmol), and triethyl orthoacetate (99%, 15.9 mL, 86.4 mmol) was heated at 145 ^{o}C for 1 h, then at 100 ^{o}C for 48 h. After cooling to room temperature, the reaction mixture was diluted with ethyl acetate (100 ml), washed with saturated aqueous sodium bicarbonate solution (30 ml), washed with water, dried over sodium sulfate, filtered, and concentrated under reduced pressure. Purification by silica gel chromatography (Gradient: 2% to 5% methanol in dichloromethane) provided the product as a light pink oil. Yield: 4.10 g, 16.2

mmol, 94%. LCMS *m/z* 254.1 (M+H). ^{1}H NMR (400 MHz, $CDCl_3$) δ 9.07 (br d, *J*=0.8 Hz, 1H), 8.36 (d, *J*=5.5 Hz, 1H), 7.15 (d, *J*=8.6 Hz, 1H), 6.89-6.97 (m, 3H), 3.90 (s, 3H), 2.42 (s, 3H), 1.94 (br s, 3H).

3-methyl-4-(2-methyl-1H-imidazo[4,5-c]pyridin-1-yl)phenol (SI-7)

Boron tribromide (1 M solution in dichloromethane, 44.1 mL, 44.1 mmol) was added drop-wise to a solution of 1-(4-methoxy-2-methylphenyl)-2-methyl-1*H*-imidazo[4,5-*c*]pyridine (**SI-6**) (3.72 g, 14.7 mmol) in dichloromethane (150 mL) at -78 ºC. The reaction mixture was stirred at -78ºC for 15 min, then the cooling bath was removed and the reaction mixture was allowed to gradually warm to room temperature. After 20 h at room temperature, the reaction mixture was recooled to -78 ºC and slowly quenched with methanol (20 ml). At this point, the cooling bath was removed; the mixture was allowed to reach ambient temperature and then stir for 15 min. Volatiles were removed *in vacuo*, methanol (100 ml) was added, and the mixture was heated at reflux for 30 min. After concentration under reduced pressure, the resulting solid was taken directly to the next step. LCMS *m/z* 240.1 (M+H).

1-[4-(furo[3,2-c]pyridin-4-yloxy)-2-methylphenyl]-2-methyl-1H-imidazo[4,5-c]pyridine (PF-8294)

A mixture of 3-methyl-4-(2-methyl-1*H*-imidazo[4,5-*c*]pyridin-1-yl)phenol (**SI-7**) (from the preceding step, ≤14.7 mmol), 4-chlorofuro[3,2-*c*]pyridine (2.37 g, 15.4 mmol) and cesium carbonate (99%, 19.3 g, 58.6 mmol) in dimethyl sulfoxide (100 mL) was heated to 140 ºC for 16 h. After cooling to room temperature, the reaction mixture was diluted with ethyl acetate (400 ml) and filtered through a pad of Celite. The filtrate was washed with water, with a 1:1 mixture of water, and with saturated aqueous sodium chloride solution (4 x 100 ml), dried over sodium sulfate, filtered, and concentrated *in vacuo*. The residue was purified by silica gel chromatography (Gradient: 2% to 10% methanol in ethyl acetate) to afford a yellow solid, which was dissolved in *tert*-butyl methyl ether (500 ml), treated with activated carbon (5 g) and heated to 40ºC. The mixture was filtered to provide a colorless solution, which was concentrated at reflux until it became cloudy (~150 mL *tert*-butyl methyl ether remaining). Upon gradual cooling to room temperature, a precipitate formed. Filtration and washing with diethyl ether afforded the product as a free-flowing white solid. Yield: 2.02 g, 5.67 mmol, 39% over 2 steps. LCMS *m/z*

357.1 (M+H). ^{1}H NMR (500 MHz, $CDCl_3$) δ 9.08 (d, *J*=1.0 Hz, 1H), 8.39 (d, *J*=5.5 Hz, 1H), 8.08 (d, *J*=5.9 Hz, 1H), 7.71 (d, *J*=2.2 Hz, 1H), 7.34-7.36 (m, 1H), 7.30 (dd, *J*=5.9, 1.0 Hz, 1H), 7.28-7.29 (m, 2H), 7.00 (dd, *J*=5.5, 1.1 Hz, 1H), 6.97 (dd, *J*=2.2, 1.0 Hz, 1H), 2.48 (s, 3H), 1.99 (br s, 3H).

PF-6142

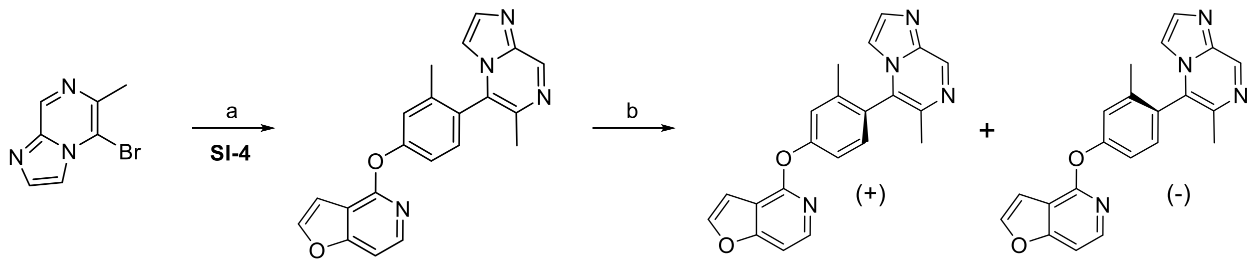

SI-8 PF-6142 SI-9

Reagents and conditions: (a) K_2CO_3, Pd(dppf)Cl_2, dioxane/water, reflux; (b) supercritical fluid chromatography.

5-[4-(furo[3,2-c]pyridin-4-yloxy)-2-methylphenyl]-6-methylimidazo[1,2-a]pyrazine (SI-8)

To a solution of 4-[3-methyl-4-(4,4,5,5-tetramethyl-1,3,2-dioxaborolan-2-yl)phenoxy]furo[3,2-*c*]pyridine (**SI-4**) (13.5 g, 38.4 mmol) in 1,4-dioxane (200 mL) and water (10 mL) were added 5-bromo-6-methylimidazo[1,2-*a*]pyrazine (see A. R. Harris et al., *Tetrahedron* **2011**, *67*, 9063-9066) (8.15 g, 38.4 mmol), potassium carbonate (15.9 g, 115 mmol) and [1,1'-bis(diphenylphosphino)ferrocene]dichloropalladium(II) (2.8 g, 3.8 mmol) at room temperature. The reaction mixture was degassed with nitrogen for 5 min, then stirred for 10 h at reflux. The mixture was cooled to room temperature and filtered. The filtrate was then concentrated *in vacuo* and purified via chromatography on silica gel (Gradient: 0% to 50% ethyl acetate in petroleum ether) to afford the product as a yellow solid. Yield: 12.4 g, 34.8 mmol, 91%. LCMS *m/z* 357.0 (M+H). ^{1}H NMR (400 MHz, CD_3OD) δ 9.02 (s, 1H), 8.00 (d, *J*=6.0 Hz, 1H), 7.93 (d, *J*=2.0 Hz, 1H), 7.79-7.80 (m, 1H), 7.48-7.51 (m, 1H), 7.44 (d, *J*=8.5 Hz, 1H), 7.41 (dd, *J*=6.0, 1.0 Hz, 1H), 7.36 (br d, *J*=2.0 Hz, 1H), 7.28 (br dd, *J*=8, 2 Hz, 1H), 7.02-7.05 (m, 1H), 2.38 (s, 3H), 2.07 (s, 3H).

(+)-5-[4-(furo[3,2-c]pyridin-4-yloxy)-2-methylphenyl]-6-methylimidazo[1,2-a]pyrazine (PF-6142)

5-[4-(Furo[3,2-*c*]pyridine-4-yloxy)-2-methylphenyl]-6-methylimidazo[1,2-*a*]pyrazine (**SI-8**) was separated into its atropenantiomers using supercritical fluid chromatography (Column: Chiralpak AD-H, 5 µm; Eluent: 3:1 carbon dioxide / methanol). **PF-6142** [designated the (+)-atropenantiomer according to its observed rotation data] was the first-eluting isomer, followed by its (-)-enantiomer (**SI-9**). **SI-9** was examined by vibrational circular dichroism (VCD) spectroscopy [Chiral*IR*™ VCD spectrometer (BioTools, Inc.)], and on the basis of this work, the absolute configuration of **SI-9** was assigned as (*R*).

PF-6142: LCMS *m/z* 357.1 (M+H). ^{1}H NMR (400 MHz, $CDCl_3$) δ 9.10 (s, 1H), 8.08 (d, *J*=5.8 Hz, 1H), 7.73 (d, *J*=1.0 Hz, 1H), 7.70 (d, *J*=2.2 Hz, 1H), 7.31-7.34 (m, 2H), 7.26-7.30 (m, 2H, assumed; partially obscured by solvent peak), 7.16-7.18 (m, 1H), 6.95 (dd, *J*=2.2, 1.0 Hz, 1H), 2.38 (s, 3H), 2.07 (br s, 3H). ^{13}C NMR (101 MHz, CHLOROFORM-*d*) δ ppm 162.17, 156.91, 155.28, 144.89, 141.68, 140.18, 139.11, 136.09, 135.49, 130.85, 127.03, 126.97, 123.13, 119.51, 113.39, 112.28, 104.33, 103.75, 20.02, 18.90.

SI-9: LCMS m/z 357.1 (M+H). 1H NMR (400 MHz, CDCl3) δ 9.10 (s, 1H), 8.09 (d, J=5.8 Hz, 1H), 7.73 (d, J=1.0 Hz, 1H), 7.70 (d, J=2.3 Hz, 1H), 7.31-7.35 (m, 2H), 7.26-7.31 (m, 2H, assumed; partially obscured by solvent peak), 7.16-7.18 (m, 1H), 6.95 (dd, J=2.2, 0.9 Hz, 1H), 2.38 (s, 3H), 2.07 (br s, 3H).

PF-2334

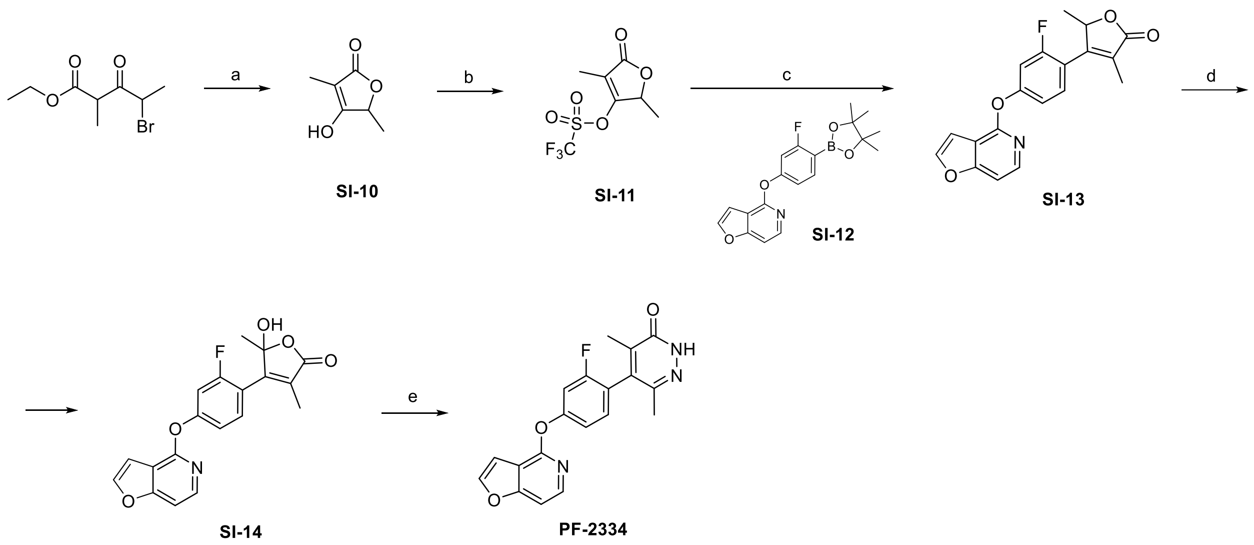

Reagents and conditions: (a) KOH, water; (b) TFA, DIPEA, dichloromethane, -20 °C to RT; (c) tetrabutylammonium chloride, $Pd(OAc)_2$, PCy_3, K_2CO_3, dioxane/water; (d) O_2, DBU, DMF, THF, 50 °C; (e) hydrazine, 1-butanol, 110°C.

4-Hydroxy-3,5-dimethylfuran-2(5H)-one (SI-10)

Methylation of ethyl 3-oxopentanoate (according to the method of D. Kalaitzakis *et al.*, *Tetrahedron: Asymmetry* **2007**, *18*, 2418-2426) produced ethyl 2-methyl-3-oxopentanoate; subsequent treatment with one equivalent of bromine in chloroform provided ethyl 4-bromo-2-methyl-3-oxopentanoate. This crude material (139 g, 586 mmol) was slowly added to a 0ºC solution of potassium hydroxide (98.7 g, 1.76 mol) in water (700 mL), and the internal reaction temperature rose to 30ºC during the addition. The reaction mixture was subjected to vigorous stirring for 4 h in an ice bath, at which point it was acidified via slow addition of concentrated hydrochloric acid. After extraction with ethyl acetate, the aqueous layer was saturated with solid NaCl and extracted three additional times with ethyl acetate. The combined organic layers were washed with saturated aqueous NaCl solution, dried over $MgSO_4$, filtered, and concentrated under reduced pressure to afford a mixture of oil and solid (81.3 g). This material was suspended in chloroform (200 ml); solids were filtered, and then washed with chloroform (2 x 50 ml). The combined filtrates were concentrated *in vacuo* and treated with a 3:1 mixture of heptane and diethyl ether (300 ml). The mixture was vigorously swirled until some of the oil began to solidify, then concentrated under reduced pressure to afford an oily solid (60.2 g). After addition

of a 3:1 mixture of heptane and diethyl ether (300 ml) and vigorous stirring for 10 min, filtration afforded the product as an off-white solid. Yield: 28.0 g, 219 mmol, 37%.

2,4-Dimethyl-5-oxo-2,5-dihydrofuran-3-yl trifluoromethanesulfonate (SI-11)

Trifluoromethanesulfonic anhydride (23.7 mL, 140 mmol) was added portion-wise to a solution of 4-hydroxy-3,5-dimethylfuran-2(5*H*)-one (**SI-10**) (15.0 g, 117 mmol) and *N*,*N*-diisopropylethylamine (99%, 24.8 mL, 140 mmol) in dichloromethane (500 mL) at -20 ºC, at a rate that maintained the internal reaction temperature below -10ºC. The reaction mixture was stirred at -20ºC, then allowed to warm gradually to 0ºC over 5 h. The reaction mixture was passed through a plug of silica gel, dried over $MgSO_4$, and concentrated in *vacuo*. The residue was suspended in diethyl ether and filtered. The filtrate was then concentrated under reduced pressure. Purification using silica gel chromatography (Gradient: 0% to 17% ethyl acetate in heptane) yielded the product as a pale yellow oil. Yield: 21.06 g, 80.94 mmol, 69%. ^1H NMR (400 MHz, $CDCl_3$) δ 5.09-5.16 (m, 1H), 1.94-1.96 (m, 3H), 1.56 (d, *J*=6.6 Hz, 3H).

4-[3-Fluoro-4-(4,4,5,5-tetramethyl-1,3,2-dioxaborolan-2-yl)phenoxy]furo[3,2-c]pyridine (SI-12)

Compound **SI-12** was synthesized using the method described for 4-[3-methyl-4-(4,4,5,5-tetramethyl-1,3,2-dioxaborolan-2-yl)phenoxy]furo[3,2-*c*]pyridine (**SI-4**), except that 4-bromo-3-fluorophenol was used in place of 4-bromo-3-methylphenol. The product was obtained as an off-white solid. Yield: 22.5 g, 63.3 mmol, 39% over 2 steps. LCMS *m/z* 356.1 (M+H). ^1H NMR (400 MHz, $CDCl_3$) δ 8.04 (d, *J*=5.9 Hz, 1H), 7.80 (dd, *J*=8.2, 6.9 Hz, 1H), 7.65 (d, *J*=2.3 Hz, 1H), 7.25 (dd, *J*=5.8, 0.9 Hz, 1H), 7.02 (dd, *J*=8.3, 2.1 Hz, 1H), 6.94 (dd, *J*=10.2, 2.1 Hz, 1H), 6.85 (dd, *J*=2.3, 1.0 Hz, 1H), 1.37 (s, 12H).

4-[2-Fluoro-4-(furo[3,2-c]pyridine-4-yloxy)phenyl]-3,5-dimethylfuran-2(5H)-one (SI-13)

A solution of 4-[3-fluoro-4-(4,4,5,5-tetramethyl-1,3,2-dioxaborolan-2-yl)phenoxy]furo[3,2-*c*]pyridine (**SI-12**) (3.20 g, 9.01 mmol) and 2,4-dimethyl-5-oxo-2,5-dihydrofuran-3-yl trifluoromethanesulfonate (**SI-11**) (2.46 g, 9.45 mmol) in 1,4-dioxane (80 ml) was purged with nitrogen for 5 min. A mixture of tetrabutylammonium chloride (99%, 127 mg, 0.452 mmol), tricyclohexylphosphine (99%, 128 mg, 0.452 mmol) and palladium(II) acetate (101 mg, 0.450 mmol) was added, followed by an aqueous solution of potassium carbonate (3 M, 9.0 mL, 27.0

mmol), and the reaction mixture was heated at 50ºC for 18 h. After cooling to room temperature, the reaction mixture was diluted with ethyl acetate, washed three times with water, washed once with saturated aqueous NaCl solution, and dried over $MgSO_4$. Filtration and removal of solvent under reduced pressure was followed by chromatographic purification on silica gel (Gradient: 15% to 50% ethyl acetate in heptane), affording the product as a tan oil that slowly solidified upon standing. Yield: 1.55 g, 4.57 mmol, 51%. LCMS *m/z* 340.3 (M+H). ^{1}H NMR (400 MHz, $CDCl_3$) δ 8.06 (d, *J*=5.9 Hz, 1H), 7.70 (d, *J*=2.2 Hz, 1H), 7.33-7.38 (m, 1H), 7.31 (dd, *J*=5.9, 1.0 Hz, 1H), 7.13-7.20 (m, 2H), 6.94 (dd, *J*=2.2, 0.9 Hz, 1H), 5.43-5.51 (m, 1H), 1.99-2.01 (m, 3H), 1.38 (d, *J*=6.6 Hz, 3H).

4-[2-Fluoro-4-(furo[3,2-c]pyridine-4-yloxy)phenyl]-5-hydroxy-3,5-dimethylfuran-2(5H)-one (SI-14)

A solution of 4-[2-fluoro-4-(furo[3,2-*c*]pyridine-4-yloxy)phenyl]-3,5-dimethylfuran-2(5*H*)-one (**SI-13**) (5.0 g, 15 mmol) in tetrahydrofuran (200 ml) and *N*,*N*-dimethylformamide (100 ml) was treated with 1,8-diazabicyclo[5.4.0]undec-7-ene (6.61 ml, 44.2 mmol) and purged with oxygen for 10 min. A slight positive pressure of oxygen was introduced into the flask and the reaction mixture was heated at 50ºC with vigorous stirring for 5 h. Upon heating, a slight additional pressure build-up was noted within the flask via examination of the rubber septum. LCMS analysis indicated approximately 6% of the starting material remaining. The flask was then cooled to room temperature, recharged with oxygen, and heated at 50ºC for an additional 18 h. The reaction was cooled to room temperature, diluted with ethyl acetate (300 ml) and washed sequentially with aqueous hydrochloric acid (0.25 M, 175 ml) and water (150 ml). The pH of the combined aqueous layers was adjusted from pH 3 to pH 4-5, and the aqueous layer was extracted with ethyl acetate (300 ml). The combined organic layers were washed with saturated aqueous NaCl solution, dried over $MgSO_4$, filtered, and concentrated *in vacuo*. Purification via silica gel chromatography (Gradient: 0% to 40% ethyl acetate in heptane) yielded the product as a white foam. Yield: 4.20 g, 11.8 mmol, 79%. LCMS *m/z* 356.4 (M+H). ^{1}H NMR (400 MHz, $CDCl_3$) δ 8.07 (d, *J*=5.8 Hz, 1H), 7.66-7.71 (m, 2H), 7.31 (br d, *J*=5.8 Hz, 1H), 7.11-7.17 (m, 2H), 6.93-6.94 (m, 1H), 3.95 (br s, 1H), 1.86-1.88 (m, 3H), 1.64 (s, 3H).

5-[2-Fluoro-4-(furo[3,2-c]pyridine-4-yloxy)phenyl]-4,6-dimethylpyridazin-3(2H)-one (PF-2334)

Anhydrous hydrazine (98.5%, 1.88 mL, 59.0 mmol) was added to a solution of 4-[2-fluoro-4-(furo[3,2-c]pyridine-4-yloxy)phenyl]-5-hydroxy-3,5-dimethylfuran-2(5H)-one (SI-14) (4.20 g, 11.8 mmol) in 1-butanol (75 ml), and the reaction mixture was heated at 110ºC for 2 h. After cooling to room temperature and stirring at this temperature for 18 h, the reaction mixture was stored in a refrigerator for 66 h. The resulting suspension was filtered to produce a gray solid, which was dissolved in hot ethanol (150 - 175 ml) and filtered through a nylon syringe filter. The filtrate was concentrated in vacuo to provide the product as a white solid. Yield: 1.30 g, 3.70 mmol, 31%. LCMS m/z 352.2 (M+H). 1H NMR (400 MHz, DMSO-d6) δ 12.89 (br s, 1H), 8.17 (d, J=2.2 Hz, 1H), 8.06 (d, J=5.8 Hz, 1H), 7.54 (br d, J=5.8 Hz, 1H), 7.38-7.46 (m, 2H), 7.25 (br dd, J=8.4, 2.2 Hz, 1H), 7.12-7.14 (m, 1H), 1.99 (s, 3H), 1.85 (s, 3H). ^{13}C NMR (101 MHz, DMSO-d_6) δ ppm 161.70, 160.70, 158.33 (d, $^{1}J_{CF}$ = 245.24 Hz), 156.15, 154.93 (d, $^{3}J_{CF}$ = 10.90 Hz), 146.65, 143.08, 141.68, 137.96, 136.95, 130.93 (d, $^{3}J_{CF}$ = 4.77 Hz), 118.89 (d, $^{2}J_{CF}$ = 17.03 Hz), 117.92 (d, $^{4}J_{CF}$ = 3.41 Hz), 112.64, 109.51 (d, $^{2}J_{CF}$ = 24.52 Hz), 104.74, 103.53, 20.05, 13.36.

PF-1119

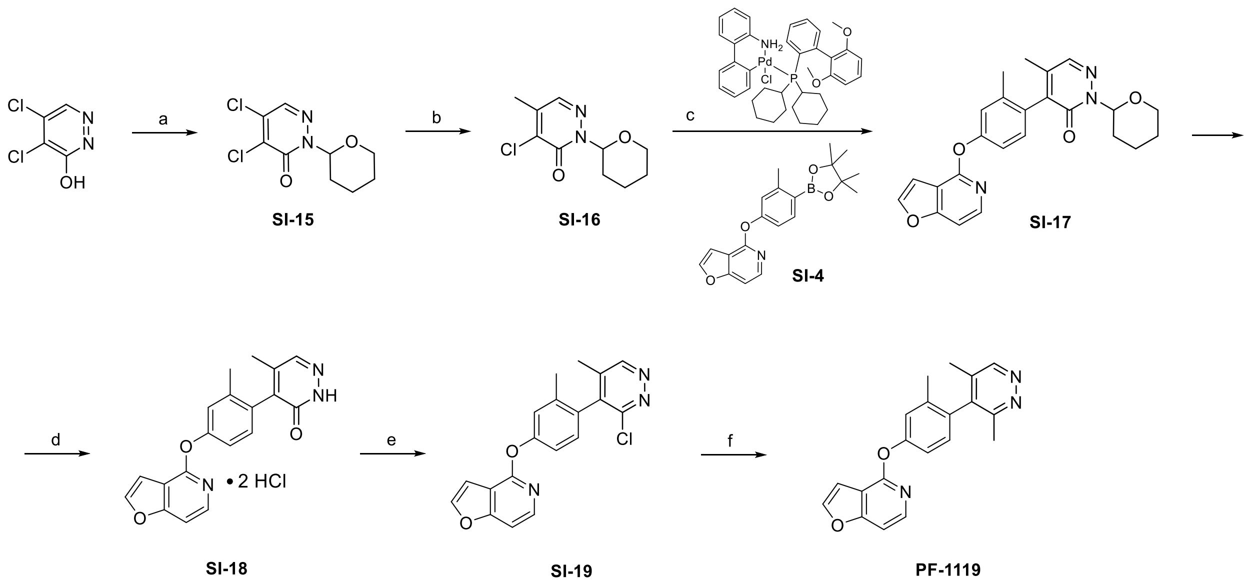

Reagents and conditions: (a) 3,4-dihydro-2H-pyran, *p*-TsOH, THF, reflux; (b) methylboronic acid, Cs_2CO_3, Pd(dppf)Cl_2, dioxane/water; (c) K_3PO_4, THF, RT; (d) HCl, dioxane; (e) $POCl_3$; (f) Me_3Al, Pd(PPh_3)$_4$, dioxane, 95 °C.

4,5-Dichloro-2-(tetrahydro-2H-pyran-2-yl)pyridazin-3(2H)-one (SI-15)

A mixture of 4,5-dichloropyridazin-3-ol (42 g, 250 mmol), 3,4-dihydro-2*H*-pyran (168 g, 2.00 mol) and *para*-toluenesulfonic acid (8.8 g, 51 mmol) in tetrahydrofuran (2 L) was refluxed for 2 d. After cooling to room temperature, the mixture was concentrated under reduced pressure. The residue was purified by chromatography on silica gel (Gradient: 3% to 5% ethyl acetate in petroleum ether) to yield the product as a white solid. Yield: 42 g, 170 mmol, 68%. ^{1}H NMR (400 MHz, $CDCl_3$) δ 7.84 (s, 1H), 6.01 (br d, *J*=11 Hz, 1H), 4.10-4.16 (m, 1H), 3.70-3.79 (m, 1H), 1.99-2.19 (m, 2H), 1.50-1.80 (m, 4H).

4-Chloro-5-methyl-2-(tetrahydro-2H-pyran-2-yl)pyridazin-3(2H)-one (SI-16)

To a mixture of 4,5-dichloro-2-(tetrahydro-2*H*-pyran-2-yl)pyridazin-3(2*H*)-one (**SI-15**) (40 g, 0.16 mol), methylboronic acid (9.6 g, 0.16 mol) and cesium carbonate (155 g, 0.476 mol) in a mixture of 1,4-dioxane (500 mL) and water (50 ml) was added [1,1'-bis(diphenylphosphino)ferrocene]dichloropalladium(II) (5 g, 7 mmol). The reaction mixture was stirred at 110 ºC for 2 h, then concentrated under reduced pressure. Purification by silica gel

chromatography (Gradient: 3% to 5% ethyl acetate in petroleum ether) provided product **SI-16** as a pale yellow solid (Yield: 9 g, 40 mmol, 25%) and its regioisomer, also as a pale yellow solid (Yield: 9.3 g, 41 mmol, 26%). **SI-16**: LCMS *m/z* 250.8 (M+Na^+). ^{1}H NMR (400 MHz, $CDCl_3$) δ 7.71 (s, 1H), 6.07 (dd, *J*=10.7, 2.1 Hz, 1H), 4.10-4.18 (m, 1H), 3.71-3.81 (m, 1H), 2.30 (s, 3H), 1.98-2.19 (m, 2H), 1.53-1.81 (m, 4H).

4-[4-(Furo[3,2-c]pyridin-4-yloxy)-2-methylphenyl]-5-methyl-2-(tetrahydro-2H-pyran-2-yl)pyridazin-3(2H)-one (SI-17)

A mixture of 4-chloro-5-methyl-2-(tetrahydro-2*H*-pyran-2-yl)pyridazin-3(2*H*)-one (**SI-16**) (457 mg, 2.00 mmol), 4-[3-methyl-4-(4,4,5,5-tetramethyl-1,3,2-dioxaborolan-2-yl)phenoxy]furo[3,2-*c*]pyridine (**SI-4**) (702 mg, 2.00 mmol) and [2'-(azanidyl-κN)biphenyl-2-yl-κC_2](chloro)[dicyclohexyl(2',6'-dimethoxybiphenyl-2-yl)-λ^5-phosphanyl]palladium (29 mg, 0.040 mmol) was subjected to three rounds of vacuum evacuation followed by introduction of nitrogen. Degassed tetrahydrofuran (4 ml) was added, followed by degassed aqueous potassium phosphate solution (0.5 M, 8.0 mL, 4.0 mmol), and the reaction mixture was stirred at room temperature for 23 h. The reaction mixture was then partitioned between ethyl acetate (20 ml) and water (8 ml), and the organic layer dried over sodium sulfate, filtered, and concentrated *in vacuo*. Purification via silica gel chromatography (Gradient: 20% to 70% ethyl acetate in heptane) afforded the product as a white solid. By NMR, this was determined to consist of a diastereomeric mixture due to the tetrahydropyranyl group. Yield: 588 mg, 1.41 mmol, 70%. LCMS *m/z* 418.0 (M+H). ^{1}H NMR (500 MHz, $CDCl_3$) δ 8.06 (d, *J*=5.9 Hz, 1H), 7.82 (d, *J*=2.8 Hz, 1H), 7.63 (d, *J*=2.3 Hz, 1H), 7.23-7.25 (m, 1H), 7.16-7.17 (m, 1H), 7.06-7.13 (m, 2H), 6.79-6.81 (m, 1H), 6.10 (dd, *J*=10.6, 2.2 Hz, 1H), 4.14-4.20 (m, 1H), 3.72-3.80 (m, 1H), 2.15-2.25 (m, 1H, assumed; partially obscured by methyl group), 2.14 and 2.15 (2 s, total 3H), 2.01-2.08 (m, 1H, assumed; partially obscured by methyl group), 2.03 and 2.04 (2 s, total 3H), 1.71-1.82 (m, 3H), 1.55-1.63 (m, 1H).

4-[4-(Furo[3,2-c]pyridin-4-yloxy)-2-methylphenyl]-5-methylpyridazin-3(2H)-one, bis-hydrochloride salt (SI-18)

4-[4-(Furo[3,2-*c*]pyridin-4-yloxy)-2-methylphenyl]-5-methyl-2-(tetrahydro-2*H*-pyran-2-yl)pyridazin-3(2*H*)-one (**SI-17**) (580 mg, 1.39 mmol) was dissolved in methanol (3 ml), treated

with a solution of hydrogen chloride in 1,4-dioxane (4 M, 5.0 ml, 20 mmol) and allowed to stir at room temperature for 3 h. Removal of solvent under reduced pressure provided the product as a pale yellow solid, presumed to be the bis-hydrochloride salt. Yield: 550 mg, 1.35 mmol, 97%. LCMS *m/z* 334.0 (M+H). ^{1}H NMR (400 MHz, DMSO-d_6) δ 13.01 (br s, 1H), 8.15 (d, *J*=2.3 Hz, 1H), 8.02 (d, *J*=5.8 Hz, 1H), 7.89 (s, 1H), 7.48 (dd, *J*=5.8, 1.1 Hz, 1H), 7.16-7.18 (m, 1H), 7.08-7.12 (m, 3H), 2.06 (br s, 3H), 1.95 (s, 3H).

4-[4-(3-Chloro-5-methylpyridazin-4-yl)-3-methylphenoxy]furo[3,2-c]pyridine (SI-19)

4-[4-(Furo[3,2-*c*]pyridin-4-yloxy)-2-methylphenyl]-5-methylpyridazin-3(2*H*)-one, bis-hydrochloride salt (**SI-18**) (550 mg, 1.35 mmol) was suspended in phosphorus oxychloride (6.0 mL, 64 mmol), and the reaction mixture was heated at 90ºC for 2 h. After removal of phosphorus oxychloride under reduced pressure, the residue was partitioned between dichloromethane (35 ml), water (10 ml), and saturated aqueous sodium bicarbonate solution (10 ml). The organic layer was dried over sodium sulfate, filtered, and concentrated *in vacuo* to yield the product as a foamy, pale amber solid. Yield: 465 mg, 1.32 mmol, 98%. LCMS *m/z* 352.0 (M+H). ^{1}H NMR (400 MHz, $CDCl_3$) δ 9.07 (s, 1H), 8.11 (d, *J*=5.8 Hz, 1H), 7.69 (d, *J*=2.3 Hz, 1H), 7.31 (dd, *J*=5.9, 0.9 Hz, 1H), 7.25-7.28 (m, 1H, assumed; partially obscured by solvent peak), 7.21-7.24 (m, 1H), 7.09 (d, *J*=8.2 Hz, 1H), 6.84 (dd, *J*=2.2, 0.8 Hz, 1H), 2.19 (s, 3H), 2.08 (br s, 3H).

4-[4-(3,5-Dimethylpyridazin-4-yl)-3-methylphenoxy]furo[3,2-c]pyridine (PF-1119)

Nitrogen was bubbled into a mixture of tetrakis(triphenylphosphine)palladium(0) (31.0 mg, 0.027 mmol) and 4-[4-(3-chloro-5-methylpyridazin-4-yl)-3-methylphenoxy]furo[3,2-*c*]pyridine (**SI-19**) (427 mg, 1.21 mmol) in 1,4-dioxane (12 ml) for 10 min. A solution of trimethylaluminum in toluene (2 M, 1.2 ml, 2.4 mmol) was added, and the reaction mixture was heated to 95ºC for 90 min, then cooled in an ice bath and treated drop-wise with methanol (12 ml) *(Caution: gas evolution.)* The mixture was filtered through Celite and the filter cake was rinsed with additional methanol (35 ml); the filtrate was concentrated *in vacuo* and purified using silica gel chromatography (Eluent: 2.5% methanol in ethyl acetate) to provide the product as a solid. Yield: 320 mg, 0.966 mmol, 80%. LCMS *m/z* 332.1 (M+H). ^{1}H NMR (500 MHz, CD_3OD) δ 9.05 (s, 1H), 7.99 (d, *J*=6.0 Hz, 1H), 7.90 (d, *J*=2.2 Hz, 1H), 7.39 (dd, *J*=5.9, 0.9 Hz, 1H),

7.26-7.27 (m, 1H), 7.19 (br dd, half of ABX pattern, *J*=8.3, 2.1 Hz, 1H), 7.15 (d, half of AB pattern, *J*=8.3 Hz, 1H), 6.94 (dd, *J*=2.2, 1.0 Hz, 1H), 2.42 (s, 3H), 2.16 (s, 3H), 2.03 (s, 3H).

PF-06421119

^{13}C NMR (101 MHz, METHANOL-d_4) δ ppm 164.04, 160.12, 158.91, 155.97, 152.98, 147.28, 142.63, 141.91, 139.28, 138.44, 132.35, 130.54, 124.37, 120.68, 114.83, 105.55, 104.84, 20.78, 19.65, 16.88.

PF-1437

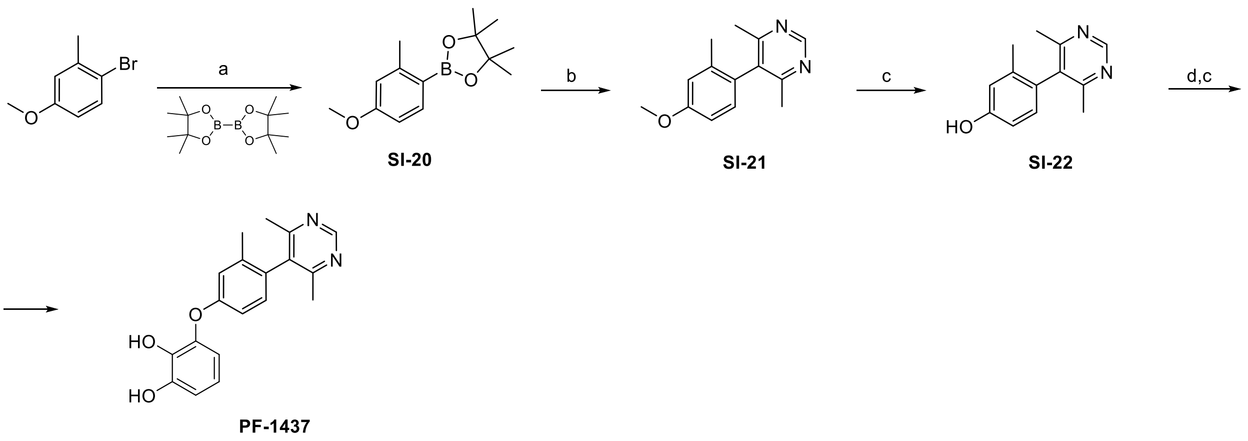

Reagents and conditions: (a) Pd(dppf)Cl_2, KOAc, dioxane; (b) 5-bromo-4,6-dimethylpyrimidine, $Pd_2(dba)_3$, PCy_3, K_3PO_4, dioxane/water; (c) BBr_3, dichloromethane, RT; (d) 2,3-dimethoxyphenyl boronic acid, $Cu(OAc)_2$, DMAP, MeCN.

2-(4-methoxy-2-methylphenyl)-4,4,5,5-tetramethyl-1,3,2-dioxaborolane (SI-20)

Compound **SI-20** was prepared from 1-bromo-4-methoxy-2-methylbenzene according to the procedure for the synthesis of 4-[3-methyl-4-(4,4,5,5-tetramethyl-1,3,2-dioxaborolan-2-yl)phenoxy]furo[3,2-*c*]pyridine (**SI-4**). The product was obtained as a solid. Yield: 15 g, 60 mmol, 80%.

5-(4-methoxy-2-methylphenyl)-4,6-dimethylpyrimidine (SI-21)

The product was prepared from 2-(4-methoxy-2-methylphenyl)-4,4,5,5-tetramethyl-1,3,2-dioxaborolane (**SI-20**) and 5-bromo-4,6-dimethylpyrimidine according to the procedure

described for preparation of **PF-8871** from **SI-4**. The product was obtained as a solid. Yield: 3.5 g, 15 mmol, 75%.

4-(4,6-Dimethylpyrimidin-5-yl)-3-methylphenol (SI-22)

Boron tribromide (3.8 mL, 40 mmol) was added drop-wise to a solution of 5-(4-methoxy-2-methylphenyl)-4,6-dimethylpyrimidine (**SI-21**) (3.0 g, 13 mmol) in dichloromethane (150 mL) at -70ºC. The reaction mixture was stirred at room temperature for 16 h, then adjusted to pH 8 with saturated aqueous sodium bicarbonate solution. The aqueous layer was extracted with dichloromethane (3 x 200 mL), and the combined organic layers were dried over sodium sulfate, filtered, and concentrated *in vacuo*. Silica gel chromatography (Gradient: 60% to 90% ethyl acetate in petroleum ether) yielded the product as a yellow solid. Yield: 1.2 g, 5.6 mmol, 43%. LCMS *m/z* 215.0 (M+H). ^{1}H NMR (400 MHz, $CDCl_3$) δ 8.98 (s, 1H), 6.89 (d, *J*=8.0 Hz, 1H), 6.86 (d, *J*=2.3 Hz, 1H), 6.80 (dd, *J*=8.3, 2.5 Hz, 1H), 2.24 (s, 6H), 1.96 (s, 3H).

3-(4-(4,6-Dimethylpyrimidin-5-yl)-3-methylphenoxy)benzene-1,2-diol (12; PF-1437)

A mixture of 4-(4,6-dimethylpyrimidin-5-yl)-3-methylphenol (**SI-22**; 428 mg, 2 mmol), 2,3-dimethoxyphenyl boronic acid (1.82 g, 10 mmol), copper (II) acetate (727 mg, 4 mmol) and DMAP (488 mg, 4 mmol) in acetonitrile (40 ml) was stirred at 80ºC for 8 h. LCMS showed formation of about 10% of the desired product. The mixture was filtered and the filtrate was concentrated *in vacuo*. The residue was purified by Combi-Flash (from 20% of EtOAc in petroleum ether to 100% of EtOAc) to yield a crude product (450 mg, 20% purity by LCMS) as a yellow solid.

The crude material was dissolved in dichloromethane (30 ml) followed by dropwise addition of boron tribromide (500 mg, 2 mmol) at -78ºC. The mixture was stirred while allowing to warm up to room temperature over 2 h. The reaction mixture was cooled again to -78ºC followed by addition of methanol (15 ml) at -78ºC. After warming to room temperature, the pH of the mixture was adjusted to ~7.5 with solid $NaHCO_3$. This mixture was filtered and the filtrate was concentrated *in vacuo*. Silica gel chromatography (Gradient: 0 to 10% methanol in dichloromethane) afforded the product as a yellow solid. Yield: 20 mg, 31%. LCMS *m/z* 323.1 (M+H) . ^{1}H NMR (400 MHz, DMSO-d_6) δ ppm 9.36 (br. s., 1 H), 8.88 (s, 1 H), 8.67 (br. s., 1 H), 7.03 (d, *J*=8.61 Hz, 1 H), 6.89 (d, *J*=2.35 Hz, 1 H), 6.77 (dd, *J*=8.22, 2.35 Hz, 1 H), 6.63 -

6.68 (m, 2 H), 6.47 - 6.52 (m, 1 H), 2.11 (s, 6 H), 1.90 (s, 3 H). ^{13}C NMR (101 MHz, DMSO-d_6) δ ppm 164.19, 157.83, 156.37, 147.22, 143.13, 137.85, 136.66, 132.16, 129.72, 128.96, 118.75, 117.76, 114.14, 112.41, 112.19, 22.26, 19.22.
