# Supplementary material for: Impaired β-arrestin recruitment and reduced desensitization by non-catechol agonists of the D1 dopamine receptor
Source: Nat Commun. 2018 Feb 14;9:674. doi: 10.1038/s41467-017-02776-7 (PMC5813016; doi:10.1038/s41467-017-02776-7)
Supplement: Supplementary file 2 — Description of Additional Supplementary Files [file 41467_2017_2776_MOESM2_ESM.pdf]

Description of Additional Supplementary Files

File Name: Supplementary Movie 1
Description: TIRFM imaging of β-arrestin-GFP membrane recruitment following administration of vehicle control. Representative live cell total internal reflection fluorescence microscopy (TIRFM) imaging of β-arrestin-GFP to the plasma membrane of U2OS cells expressing human D1R. The movie is a time lapse that shows β-arrestin-GFP at the plasma membrane before and for 10 min after addition of vehicle control.

File Name: Supplementary Movie 2
Description: TIRFM imaging of β-arrestin-GFP membrane recruitment following administration of dopamine. Representative live cell total internal reflection fluorescence microscopy (TIRFM) imaging of β-arrestin-GFP to the plasma membrane of U2OS cells expressing human D1R. The movie is a time lapse that shows β-arrestin-GFP at the plasma membrane before and for 10 min after addition of dopamine (10 μM). Appearance of puncta with increased intensity is indicative of β-arrestin recruitment.

File Name: Supplementary Movie 3
Description: TIRFM imaging of β-arrestin-GFP membrane recruitment following administration of SKF-81297. Representative live cell total internal reflection fluorescence microscopy (TIRFM) imaging of β-arrestin-GFP to the plasma membrane of U2OS cells expressing human D1R. The movie is a time lapse that shows β-arrestin-GFP at the plasma membrane before and for 10 min after addition of SKF-81297 (10 μM). Appearance of puncta with increased intensity is indicative of β-arrestin recruitment.

File Name: Supplementary Movie 4
Description: TIRFM imaging of β-arrestin-GFP membrane recruitment following administration of PF-6142. Representative live cell total internal reflection fluorescence microscopy (TIRFM) imaging of β-arrestin-GFP to the plasma membrane of U2OS cells expressing human D1R. The video is a time lapse that shows β-arrestin-GFP at the plasma membrane before and for 10 min after addition of PF-6142 (10 μM). Minimal increase in puncta with increased intensity is indicative of weak or no β-arrestin recruitment.

File Name: Supplementary Movie 5
Description: Dopamine docked in the D1R orthosteric site with full view, 360° rotations and zoom-in. Computationally-derived low-energy binding pose of dopamine docked in orthosteric binding site of human D1 receptor homology model. Movie rotates and zooms on a static binding mode. Predicted contacts between catechol hydroxyl's and S202 and S198 are visible as is interaction between secondary amine and D103.

File Name: Supplementary Movie 6
Description: PF-6142 docked in the D1R orthosteric site with full view, 360° rotations and zoom-in. Computationally-derived low-energy binding pose of PF-6142 docked in the orthosteric binding site of the human D1 receptor homology model. Movie rotates and zooms

on a static binding mode. Predicted contacts between imidazopyrazine S188/L190 on ECL2 are visible.

File Name: Supplementary Movie 7
Description: Molecular dynamics simulation of PF-8871 binding mode at the D1R. 50 ns molecular dynamic simulation of PF-8871 in the orthosteric binding site of the human D1 receptor homology model. The simulation includes solvation and lipid bilayer. Note that the ligand remains within the initial binding site for the entire simulation, suggesting that the proposed binding mode is energetically stable to normal protein conformational variation.

File Name: Supplementary Movie 8
Description: PF-8871 docked in the D1R orthosteric site with full view, 360° rotations and zoom-in. Computationally-derived low-energy binding pose of PF-8871 docked in the orthosteric binding site of human D1 receptor homology model. Movie rotates and zooms on a static binding mode. Predicted contacts between dimethylpyrimidine and S188/L190 on ECL2 are visible.

File Name: Supplementary Movie 9
Description: PF-1437 docked in the D1R orthosteric site with full view, 360° rotations and zoom-in. Computationally-derived low-energy binding pose of PF-1437 docked in the orthosteric binding site of the human D1 receptor homology model. Movie rotates and zooms on a static binding mode. Predicted hydrogen-bonding contacts between catechol hydroxyls and S198 and S202 are visible.

File Name: Supplementary Movie 10
Description: Molecular dynamics simulation of PF-1437 binding mode at the D1R. 50 ns molecular dynamic simulation of PF-1437 in the orthosteric binding site of the human D1 receptor homology model. The simulation includes solvation and lipid bilayer. Note that the ligand remains within the initial binding site for the entire simulation. Optimal or near-optimal hydrogen bonding distance and angle between catechol hydroxyls and S202 and S198 is maintained throughout the entire simulation, suggesting proposed binding mode is energetically stable to normal protein conformational variation.

File Name: Supplementary Movie 11
Description: Molecular dynamics simulation of PF-1437 binding mode at the D1R, zoomed to orthosteric binding pocket. Taken from same simulation as Supplementary Movie 10, this is a closer view of orthosteric binding site of human D1 receptor homology model with PF-1437 modeled and bound. The simulation includes solvation and lipid bilayer. Note that the ligand remains within the initial binding site for the entire simulation. Optimal or near-optimal hydrogen bonding distance and angle between catechol hydroxyls and S202 and S198 is maintained throughout the entire simulation, suggesting proposed binding mode is energetically stable to normal protein conformational variation.
